# A Methionine Allocation Nanoregulator for the Suppression of Cancer Stem Cells and Support to the Immune Cells by Epigenetic Regulation

**DOI:** 10.1002/advs.202415207

**Published:** 2025-02-22

**Authors:** Boyu Su, Qinjun Chen, Xuwen Li, Mingzhu Fang, Yu Wang, Haolin Song, Haoyu You, Zheng Zhou, Yuxing Wu, Zhenhao Zhao, Yun Chen, Hongrui Fan, Chufeng Li, Chen Jiang, Tao Sun

**Affiliations:** ^1^ Department of Pharmaceutics School of Pharmacy Fudan University Key Laboratory of Smart Drug Delivery Ministry of Education State Key Laboratory of Medical Neurobiology and MOE Frontiers Center for Brain Science Shanghai 201203 China; ^2^ Department of Digestive Diseases National Regional Medical Center Binhai Campus of the First Affiliated Hospital Fujian Medical University Fuzhou 350212 China; ^3^ Quzhou Fudan Institute Quzhou 324003 China

**Keywords:** cancer therapy, epigenetic, immune, methionine, oxaliplatin, siRNA

## Abstract

Epigenetic dysregulation is prevalent in human cancers, affecting gene expression and metabolic patterns to meet the demands of malignant evolution and abnormal epigenetic processes, and resulting in a protumor immune microenvironment. Tumors require a steady supply of methionine for maintaining epigenetic flexibility, which is the only exogenous precursor of methyl donor S‐adenosylmethionine for methylation, crucial for their resistance to therapies and survival in a nutrient‐deficient microenvironment. Thus, tumor cells upregulate the Lat4 transporter to compete and deprive methionine in the microenvironment, sustaining their malignant phenotypes and also impairing immune cell functions. Addressing this methionine addiction is the key to overcoming drug resistance and improving immune response. Despite the challenge of lacking specific Lat4 inhibitors, an oxaliplatin prodrug crosslinked fluorinated polycation/anti‐Lat4 small interfering RNA complex nanoregulator (AS‐F‐NP) has been designed and developed here. This nanoregulator restricted the greedy methionine uptake of tumor cells by knocking down Lat4, which in turn inhibited the malignant evolution of the tumor while restoring the viability and function of tumor‐infiltrating immune cells.

## Graphical abstract

A nanoregulator interfering with intratumoral methionine competition is designed to deliver oxaliplatin (IV) prodrugs and anti‐Lat4 small interfering RNA to tumor cells. This Pt (IV)‐crosslinked reduction‐sensitive nanoregulator progressively releases drugs within tumor cells and induces immunogenic cell death. Ultimately, it inhibits malignant tumor evolution and restores immune effector cell function based on epigenetic regulation by interfering with tumor methionine uptake.

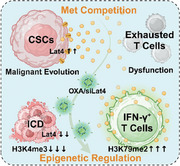

## 1 Introduction

Epigenetic dysregulation is a hallmark of almost all human cancers, directly impacting the gene expression and phenotypes of tumor cells and driving them into abnormal metabolic patterns to meet the metabolic demands of this dysregulated epigenetic process.^[^ ^1^, ^2^ ^]^ Due to the flexible plasticity of epigenetics, characterized by reversible modifications of epigenetic markers, a sufficient pool of raw materials is required to maintain the dynamic balance of epigenetic regulation.^[^ ^2^ ^]^ This is also a prerequisite for tumor cells to develop resistance to therapeutic stimuli or transform into highly resistant phenotypes.^[^ ^3^ ^]^ Furthermore, the frantic competition for nutrients driven by this abnormal metabolic pattern also affects the tumor microenvironment and immune cells, creating a microenvironment characterized by nutrient deficiency, leading to impaired immune cell function and tumor survival.^[^ ^4^, ^5^, ^6^ ^]^ Therefore, identifying and targeting the crucial point in abnormal metabolism resulting from epigenetic dysregulation is essential for reversing tumor therapy resistance and reshaping the suppressive tumor immune microenvironment.

The epigenetic dysregulation in tumors presents as abnormal epigenetic modifications on DNA or histones, determining the activation or inhibition of gene expression that promotes tumor progression, among which methylation modifications on histones or DNA are closely associated with the tumor malignant evolution, including the maintenance of cancer stem cell phenotypes, resistance to chemotherapy, and potentials of invasion and metastasis.^[^ ^7^ ^]^ Additionally, methylation modifications are essential for the activation and differentiation of immune cells, especially for the acquisition of effector function by CD8^+^ T cells.^[^ ^8^ ^]^ However, the active methyl groups required for methylation come solely from methionine, an essential amino acid that must be obtained externally. Consequently, tumor cells develop an addictive uptake and abnormal metabolism pattern for methionine, providing a crucial metabolic basis for maintaining epigenetic methylation dysregulation.^[^ ^5^, ^9^ ^]^ Tumor cells significantly upregulate Lat4, the transporter mainly responsible for methionine transport, compared to other cells, to enhance their methionine uptake competitiveness. This is also supported by using GEPIA tools and the immunofluorescence images of patient‐derived sections (Figure S1, Supporting Information). As a result, on one hand, the abundant methionine provides tumor cells with ample methyl donors, ensuring their flexible epigenetic plasticity, including maintaining a stem cell‐like phenotype to resist chemotherapy.^[^ ^10^ ^]^ On the other hand, this excessive methionine uptake severely depletes methionine in the tumor microenvironment.^[^ ^11^, ^12^ ^]^ Tumor‐infiltrating immune cells may experience reduced vitality or functional loss due to inadequate methionine intake, further resulting in poor immune response efficacy and suppressive immune microenvironment (Figure S2, Supporting Information). Therefore, the voracious methionine uptake by tumor cells may be a central factor in the failure of antitumor treatments. Cutting off this addiction to methionine uptake by tumor cells is crucial for reversing tumor epigenetic dysregulation, overcoming drug resistance, restoring the balance of methionine distribution in the microenvironment, and thus restoring the effector function of immune cells.

Due to the lack of suitable small molecule inhibitors targeting Lat4, gene‐based intervention, such as small interfering RNA (siRNA), represent a promising strategy. However, gene therapy relies on delivery vectors. The efficacy of gene therapy is highly dependent on the characteristics of the delivery vector, such as its integrity in systemic circulation and the rapid release upon entering target cells.^[^ ^13^ ^]^ This necessitates the incorporation of responsive modules in the vector that can trigger morphological changes in response to environmental stimuli.^[^ ^14^, ^15^ ^]^ Oxaliplatin (OXA), an immunogenic chemotherapeutic agent that can be readily modified into prodrugs with responsive linker characteristics, emerges as an optimal candidate for this crucial component.^[^ ^16^, ^17^ ^]^ Beyond its suitability in siRNA‐vector construction and responsive drug release, the ability of OXA to induce immunogenic cell death across a wide range of cancer types can also serve as an ignitor for antitumor immunotherapy, activating antigen‐presenting cells (APCs) and initiating immune response programs.^[^ ^18^ ^]^ Complementarily, concurrently with antigen presentation and immune cell recruitment, siLat4 suppresses the tumor cell's excessive depletion of methionine in the microenvironment, creating a conducive environment for immune effector cells to kill tumors. On the other hand, methionine metabolism restriction also impedes the evolution of cancer stem cell phenotypes, providing a better context for OXA to exert its tumor‐killing effects. Therefore, a therapeutic strategy combining these two for co‐delivery is a very promising option.

**Scheme 1:**
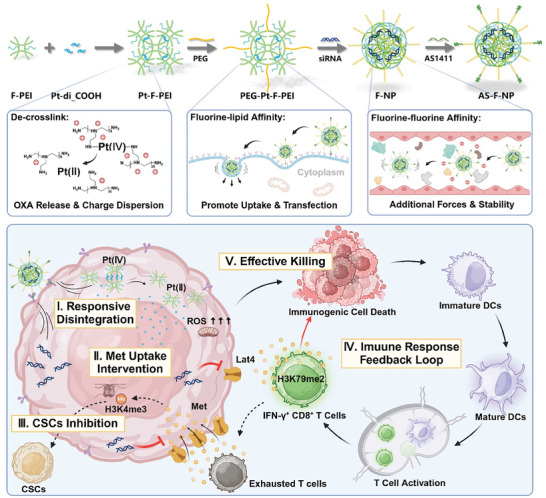
Schematic illustration of nanoregulator preparation, function and its mediation of methionine reallocation leading to restoration of an intact immune response feedback loop and synergistic tumor suppression.

Here, we have devised a fluorinated polycation/siRNA complex named AS‐F‐NP working as a methionine allocation nanoregulator for combination therapies (Scheme 1). This nanoregulator features a fluorinated low molecular weight polyethyleneimine crosslinked with OXA grafted with succinic anhydride, enabling efficient encapsulation and stable compression of siRNAs, enhancing their circulation stability and transfection efficiency. Additionally, the nanoregulator can degrade and release drugs in response to reductive substances in tumor cells that are higher than normal cells, thereby avoiding systemic chemotherapy toxicity and accumulation toxicity of high molecular weight polycations in vivo. By strategically aligning with the regulation of methionine uptake and immunogenic chemotherapy, this nanoregulator effectively modulates methionine distribution in tumors by silencing Lat4 expression in tumor cells. As a result, this intervention inhibits cancer stem cells, restores the function of tumor‐infiltrating immune cells, promotes immune response efficacy, and ultimately impedes the progression of two lung metastatic tumor models.

## 2 Results

### 2.1 Preparation and Characterization of AS‐F‐NP

**Figure 1:**
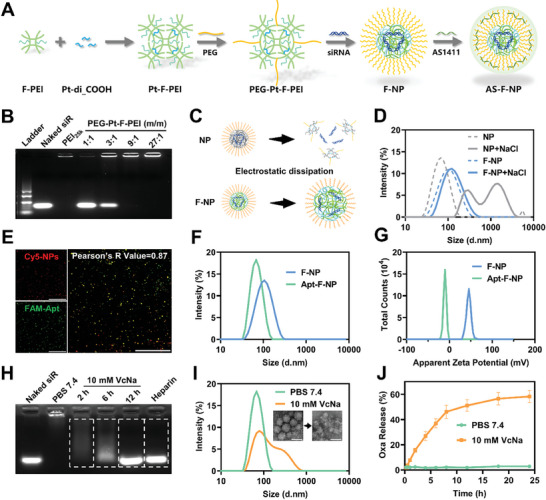
Preparation and characterization of AS‐F‐NP. A) Schematic illustration of the preparation of AS‐F‐NP. B) Agarose gel electrophoresis of nanoparticles with different polymer/siRNA ratios. C) Schematic illustration of fluorine–fluorine affinity to enhance nanoparticle stability (NP, fluorine‐free nanoparticles; F‐NP fluorine‐containing nanoparticles). D) Size distribution changes of NP or F‐NP before and after disruption of electrostatic interactions (NP, fluorine‐free nanoparticles; F‐NP fluorine‐containing nanoparticles). E) CLSM images of Apt‐F‐NP (scale bar = 5 µm). F) Size distribution and G) Zeta potential distribution before and after surface modification of F‐NP by DNA aptamer. H) Agarose gel electrophoresis of NPs after incubation for different times with 10 mM VcNa (lane 1, naked siRNA; lane 2, Apt‐F‐NP in PBS 7.4 for 12 h; lane 3, Apt‐F‐NP in 10 mM VcNa for 2 h; lane 4, Apt‐F‐NP in 10 mM VcNa for 6 h; lane 5, Apt‐F‐NP in 10 mM VcNa for 12 h; lane 6, Apt‐F‐NP in PBS 7.4 with 15 µM heparin). I) DLS results and TEM images of Apt‐F‐NP after 12 h of incubation with PBS 7.4 or 10 mM VcNa (scale bar = 100 nm). J) Release curve of OXA in Apt‐F‐NP under different conditions (*n* = 4, data were presented as the means ± SEM).

Stable protection and efficient delivery of specific vectors are fundamental for the effective function of siRNA.^[^ ^13^ ^]^ Polyethyleneimine (PEI) with a molecular weight of 25 kDa (PEI_25k_) is the gold standard for in vitro siRNA transfection, but its strong positive charge and non‐degradable high molecular weight limit its use for in vivo transfection.^[^ ^19^ ^]^ While low molecular weight PEI (PEI_1.2k_) avoids the problem of body‐accumulated toxicity, it struggles to compact short segments of siRNA to the appropriate particle size for prolonged circulation and barrier‐penetration. Hence, the optimal approach is to crosslink low molecular weight PEI with degradable crosslinkers to create a highly positively charged polycation carrier. Moreover, considering the instability of OXA in the in vivo water environment, its tendency to cause kidney toxicity and allergic reactions, as well as its reliance on membrane transport proteins for intracellular entry, OXA was firstly oxidized to a tetravalent platinum prodrug and then was carboxylated to produce a linker (Pt‐di_COOH) for crosslinking PEI_1.2k_ (Figures S3–S7, Supporting Information). In addition, the PEI_1.2k_ was premodified with fluorination, which has been reported to enhance the transfection efficiency of siRNA.^[^ ^20^ ^]^ And the additional stabilizing force provided by the fluorination modification was also found in our subsequent studies. Maleimide‐polyethylene glycol was then modified onto the crosslinked polycation polymer (PEG‐Pt‐F‐PEI) for subsequent surface hydrophilic ligand modification‐mediated charge reversal (**Figure** **1**A).

By simply mixing and vortex, PEG‐Pt‐F‐PEI can electrostatically wrap and compress siRNA to form nanoparticles with a size of ≈100 nm (F‐NP). Through agarose gel electrophoresis (Figure 1B), we confirmed that the polycationic carrier could completely compress siRNA at an N/P ratio of 10, with an encapsulation efficiency of 98.57 ± 0.51% (Figure S8, Supporting Information). Furthermore, a crosslinked polycationic polymer without fluorination was synthesized as a control to study the role of fluorination in the stability and antistatic interference capabilities of nanoparticles (Figure S9, Supporting Information). Upon the addition of 3 M NaCl aqueous solution to shield electrostatic forces, severe dispersal of the NP nanoparticle size distribution was observed, while the F‐NP size only slightly increased, with a polydispersity index (PDI) still <0.3 (Figure 1C,D). This suggests that, besides electrostatic forces, the unique fluorine–fluorine affinity significantly contributes to the overall stability of the nanoparticles, offering potential for stable in vivo delivery in circulation. Since F‐NP possesses a high surface charge and lacks selective targeting capability for tumors, the nanoparticle surface was modified in situ with AS1411, a DNA aptamer that specifically recognizes the nucleolins exposured on the surface of tumor cells, using gentle covalent coupling between maleimide (Mal) and thiol (–SH).^[^ ^21^ ^]^ The differences in electrophoretic displacement in agarose gel electrophoresis reveal the occurrence of this covalent coupling reaction (Figure S10, Supporting Information). As shown in Figure 1E, aptamer and siRNA were labeled with fluorescence groups FAM and Cy5, respectively, and confirmed the success of this in situ modification by confocal laser scanning microscope (CLSM). Due to the additional compaction effect between the aptamer and the positively charged nanoparticles, after surface modification (AS‐F‐NP), the nanoparticle size slightly decreased (Figure 1F), and the surface became weakly negative (Figure 1G), enhancing the stability of the nanoparticles in circulation. Stability experiments in PBS and PBS containing FBS for seven days confirmed this (Figure S11, Supporting Information).

The stable protection and delivery of AS‐F‐NP provide a strong guarantee to prevent premature leakage and off‐target toxicity. However, successful tumor treatment also hinges on the precise and controllable drug release of the delivery system. Therefore, the reductive responsiveness and drug release behavior of AS‐F‐NP were further evaluated. As shown in Figure 1H, incubating with a 10 mM sodium ascorbate solution, which simulates the reducing environment within tumor cells, depicted nearly complete release of siRNA from the polycationic carrier compressing it by 12 h. At this time, dynamic light scattering (DLS) and transmission electron microscopy (TEM) pointed to significant changes in nanoparticle size and morphology (Figure 1I). Similarly, amid different incubation times, OXA exhibited responsive release behavior under reducing conditions only (Figure 1J). These results collectively indicate that AS‐F‐NP has the potential to respond to high levels of reducing substances in tumor cells, degrade, and release drugs.

### 2.2 Cellular Uptake and Tumor Targeting

**Figure 2:**
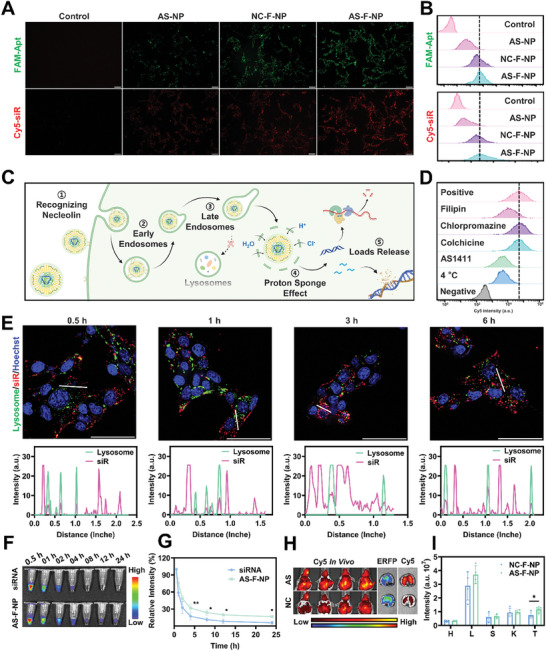
Cellular uptake and in vivo behavior of AS‐F‐NP. A) Fluorescence microscopy and B) FACS analysis of B16‐F10 cell uptake of different nanoparticles (AS‐NP, fluorine‐free nanoparticles; NC‐F‐NP, scramble‐aptamer surface modified fluorinated nanoparticles; AS‐F‐NP, AS1411 surface modified fluorinated nanoparticles; scale bar = 100 µm). C) Schematic illustration of cellular uptake and in vivo behaviors of AS‐F‐NP. D) FACS analysis of the possible AS‐F‐NP endocytosis pathway by applying different inhibitors. E) CLSM images of the intracellular behavior of AS‐F‐NP (scale bar = 50 µm) and the semiquantification results of the white line in images. F) Representative fluorescence images and G) semiquantification results of fluorescent intensity in blood of mice at different time after treatment with free Cy5‐siRNA or AS‐F‐NP/Cy5‐siRNA (*n* = 4, data were presented as the means ± SD, two‐way ANOVA, Sidak's multiple comparisons test, *p < 0.1, **p < 0.01, ***p < 0.001). H) Representative IVIS images and I) quantification results of fluorescent intensity distribution in different organs (*n* = 4, data were presented as the means ± SD, two‐tailed *T*‐test, *p < 0.1).

Cellular uptake is the first step in transfection of siRNA and the delivery of OXA in vitro, with its efficiency directly impacting gene silencing and chemotherapy efficacy. Hence, the aptamer and siRNA were labeled with FAM and Cy5, deploying AS‐NP without fluorination and NC‐F‐NP with an aptamer constructed of a random sequence as control groups. Upon incubation with B16‐F10 tumor cells, fluorescence microscopy and fluorescence‐activated cell sorting (FACS) were utilized to assess the intensity of the two fluorescence signals inside tumor cells. As shown in **Figure** **2**A,B and Figure S12 (Supporting Information), the results unveiled a profound improvement in nanoparticle uptake efficiency due to fluorination, with surface modification by AS1411 further contributing to cellular uptake efficiency to some extent. Moreover, the consistency of the trends in the two fluorescence signals among the different groups validated the integrity of AS‐F‐NP in the cellular uptake process (Figure 2B). Given the widespread expression of nucleolin on various tumor cell surfaces, similar results were found in 4T1 cells (Figure S13, Supporting Information). To delve into the cellular uptake route and mechanism of AS‐F‐NP, B16‐F10 cells were preprocessed with different inhibitors or conditions before incubated with Cy5 labeled AS‐F‐NP. FACS results unveiled that free AS1411 considerably inhibited cellular uptake through competitive binding (Figure 2D). Furthermore, filipin, an inhibitor of caveolin‐mediated endocytosis, and low‐temperature conditions significantly inhibited uptake, whereas clathrin‐mediated endocytosis and macropinocytosis inhibitors did not (Figure 2D). The findings suggest that AS‐F‐NP binds to proteins on the cell membrane via AS1411 and enters the cell through an energy‐dependent caveolin‐mediated endocytosis pathway, potentially evading the late endocytic pathway leading to lysosomes.^[^ ^22^ ^]^ The abundant primary and secondary amine groups on PEI could trigger the “proton sponge” effect during the transition from early endosomes to late endosomes, facilitating AS‐F‐NP entry into the cytoplasm, a notion confirmed in subsequent confocal laser scanning microscopy (CLSM) results (Figure 2E). To further demonstrate this, we performed continuous imaging recordings of intracellular fixed regions by time‐lapse imaging of CLSM, as shown in Video S1,2. After incubation of AS‐F‐NP with B16‐F10 cells, siRNA and lysosomes barely co‐localized within 6 h, implying potential for enhancing transfection efficiency by avoiding large amounts of siRNA entering lysosomes and risking degradation.

Results of in vitro cellular uptake and intracellular process suggest that AS‐F‐NP possesses the potential to enter tumor cells efficiently after reaching the tumor site and protect the loads from degradation. Nevertheless, the in vivo therapeutic efficacy of siRNA is restricted by clearance from circulation before reaching the tumor and random systemic distribution. By administering equal doses of Cy5‐labeled siRNA and AS‐F‐NP/Cy5‐siRNA via the tail vein to healthy mice and measuring the fluorescence intensity of blood at different time, it was observed that AS‐F‐NP effectively slowed down siRNA clearance from systemic circulation compared to free siRNA (Figure 2F,G). Additionally, compared with NC‐F‐NP, AS‐F‐NP exhibited enhanced accumulation mediated by AS1411 in 4T1 lung metastatic tumor tissues (Figure 2H,I and Figure S14, Supporting Information). This was also supported in B16‐F10 lung metastatic tumor‐bearing mouse models (Figure S15, Supporting Information). Therefore, fluorination and AS1411 modification enabled AS‐F‐NP to stably protect loads upon entering circulation and accumulate at tumor sites, revealing potential for effective antitumor therapy.

### 2.3 Intense Immunogenic Cell Death Induced by AS‐F‐NP

**Figure 3:**
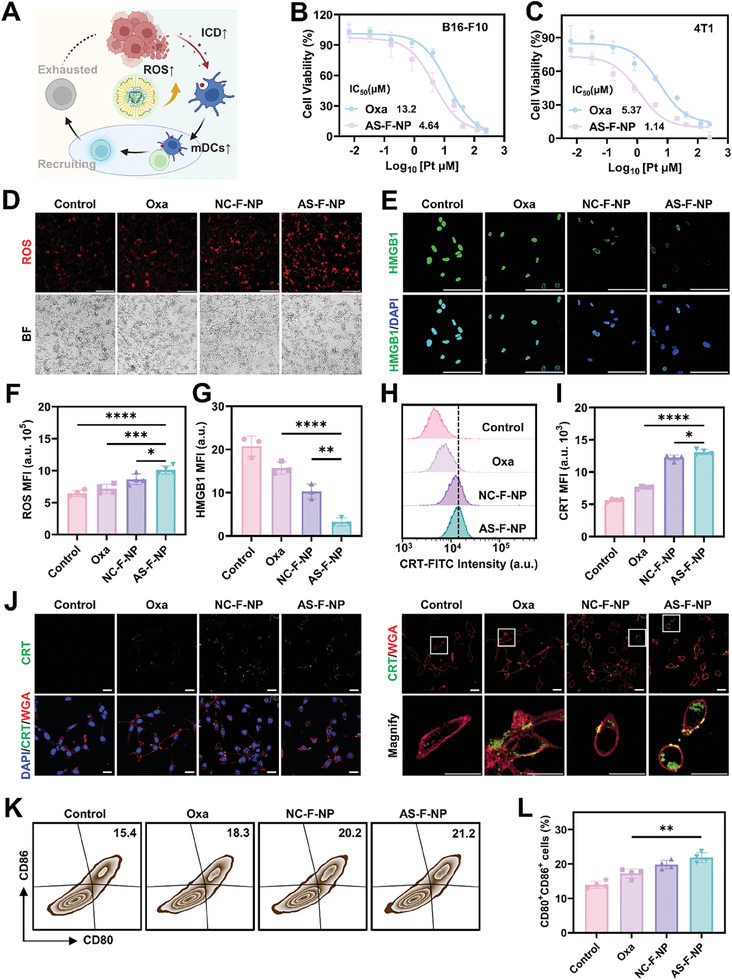
In vitro ICD inducing effect of AS‐F‐NP. A) Schematic illustration of the experimental program. In vitro antiproliferative effects of OXA and AS‐F‐NP on B) B16‐F10 tumor cells and C) 4T1 tumor cells. D) Fluorescence microscopy and F) quantification results of the mean fluorescent intensity of ROS probes in B16‐F10 cells after different treatments (scale bar = 200 µm). E) CLSM images and G) quantification results of the mean fluorescent intensity of HMGB1 in B16‐F10 cells after different treatments (scale bar = 100 µm) Data are presented as means ± SD. H) FACS analysis and I) quantification results of B16‐F10 cells with CRT membrane ectasia after different treatments. Data are presented as means ± SD (*n* = 4). J) CLSM images of CRT membrane ectasia in B16‐F10 cells (scale bar = 20 µm). K) FACS analysis and L) quantification results of DCs maturation after incubated with supernatants of B16‐F10 cells treated with different drugs/formulation. Data are presented as means ± SD (*n* = 4, one‐way ANOVA, Tukey's multiple comparisons test, *p < 0.1, **p < 0.01, ***p < 0.001, ****p < 0.0001).

OXA can inhibit the growth of tumor cells and activate immune response by inducing immunogenic cell death (ICD) to eliminate tumor cells further (**Figure** **3**A). ICD is triggered by mitochondrial DNA damage and the concomitant increase in intracellular ROS, which subsequently triggers endoplasmic reticulum stress, membrane translocation of calreticulin (CRT) and release of high mobility group box 1 (HMGB1) proteins and ATP. Thus, disruption of intracellular redox balance and elevation of ROS are crucial for inducing ICD. Designed as an oxidized platinum (IV), AS‐F‐NP responds to intracellular reducing environment, releasing OXA while consuming the reducing substances, thereby enhancing the efficiency of inducing ICD. the enhanced growth‐inhibitory effects of AS‐F‐NP on both B16‐F10 and 4T1 tumor cell lines were initially validated (Figure 3B,C). Subsequent analysis through fluorescence microscopy and FACS confirmed that compared to free OXA, both prodrug‐crosslinked nanoparticles induced higher levels of intracellular ROS in B16‐F10 tumor cells, with AS‐F‐NP exhibiting the highest intracellular ROS levels (Figure 3D, F and Figure S16, Supporting Information). Comparable results were observed in 4T1 tumor cells (Figure S17, Supporting Information). As shown in Figure 3E,G–J, and Figure S18, further evaluation of downstream ICD characteristics in B16‐F10 cells—CRT translocation to the membrane and HMGB1 release from the cell nucleus—revealed that both prodrug‐crosslinked nanoparticles elicited a stronger ICD response than free OXA, a result also corroborated in 4T1 cells (Figure S19, Supporting Information). As an initial trigger for immune response activation, the most compelling evidence of successfully induced ICD is the activation of dendritic cells (DCs). To that end, we isolated and induced bone marrow‐derived DCs from healthy C57 mice, incubating the supernatant of B16‐F10 cells in different groups with the bone marrow derived dendritic cells (BMDCs) and analyzing the proportion of activated DCs (CD86^+^CD80^+^ DCs) using FACS. The outcomes showed significant ICD in B16‐F10 cells post‐treatment with AS‐F‐NP, yielding the highest proportion of mature DCs (Figure 3K,L). Based on the above results, the capacity of AS‐F‐NP to induce intense ICD and activate DCs underscores its potential for effective antitumor therapy.

### 2.4 Immune Cells Rehabilitation and CSCs Elimination

**Figure 4:**
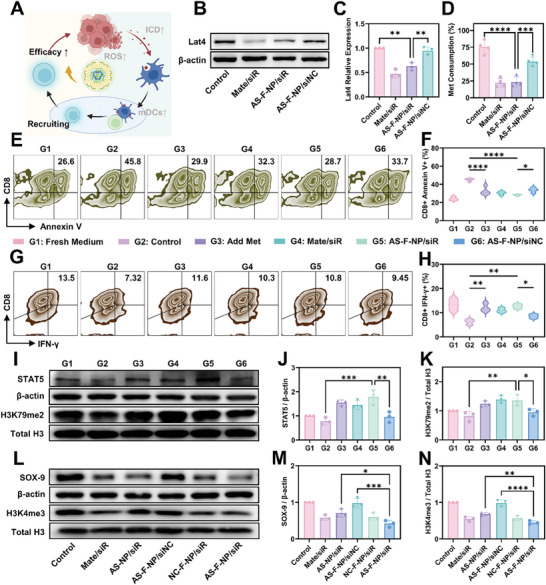
Effects of in vitro methionine restriction and epigenetic regulation of AS‐F‐NP. A) Schematic illustration of methionine restriction and downstream effects. B) Western blot assay and C) semiquantification analysis of Lat4 expression in B16‐F10 cells after different treatment. Data are presented as means ± SD (*n* = 3) (D) Methionine consumption rate of B16‐F10 cells after different treatments compared to fresh medium. Data are presented as means ± SD (*n* = 4). E) FACS analysis and F) quantification results of the apoptosis of CD8+ T cells after incubated with different medium or supernatants from B16‐F10 cells treated with different formulation. G) FACS analysis and H) quantification results of the function evaluation of CD8+ T cells after incubated with different medium or supernatants from B16‐F10 cells treated with different formulation. I) Western blot assay and semiquantification analysis of J) STAT5 and K) H3K79me2 expression in CD8+ T cells after incubated with different medium or supernatants from B16‐F10 cells treated with different formulation. L) Western blot assay and semiquantification analysis of M) SOX‐9 and N) H3K4me3 expression in 4T1 cells after treated with different formulations. Data are presented as means ± SD (*n* = 4, one‐way ANOVA, Tukey's multiple comparisons test, *p < 0.1, **p < 0.01, ***p < 0.001, ****p < 0.0001).

Methionine deprivation and competition as one of the main characteristics of the tumor microenvironment severely impacts the vitality and function of tumor‐infiltrating immune cells. For example, due to the crucial role of methionine in epigenetic regulation, T cells require sufficient methionine and methylation modification on histone lysine (H3K79me2, dimethylation of lysine 79 on histone H3) to initiate the signal transducer and activator of transcription 5 (STAT5) pathway and IFN‐γ secretion. However, tumor cells significantly upregulate Lat4 to aggressively compete for methionine, meeting their own malignant phenotype maintenance needs while compromising the metabolism and function of T cells. Therefore, we hope that AS‐F‐NP loaded with siLat4 can efficiently activate ICD while also restoring the function of tumor‐infiltrating immune effector cells by breaking the imbalance of methionine allocation, reversing the immunosuppressive microenvironment, and generating potent antitumor immune killing (**Figure** **4**A). We first demonstrated through Western blotting (WB) that AS‐F‐NP can effectively transfect and knock down the expression levels of Lat4 on B16‐F10 cells (Figure 4B,C). Correspondingly, there was a significant reduction in methionine consumption in the culture medium with 30 µM methionine to simulate the plasma concentration (Figure 4D). Similar results were also obtained in 4T1 cells (Figures S20 and S21, Supporting Information). Subsequently, focusing on the main immune effector cells, we isolated and induced activated CD8^+^ T cells from the spleens of C57 mice, which were then incubated with fresh medium or supernatants from tumor cells subjected to different treatments. According to FACS results in Figure 4E–H, fresh medium containing sufficient methionine maintained a low level of apoptosis and a high level of IFN‐γ^+^ T cells. In contrast, untreated tumor cell supernatants induced apparent apoptosis and functional impairment of CD8^+^ T cells, which could be restored by additional supplementation of methionine. The supernatant of tumor cells treated with AS‐F‐NP loaded with siLat4 showed a reduction in CD8^+^ T cell apoptosis and restoration of effector function similar to the commercial transfection reagent Mate, which was not observed in the supernatant treated with AS‐F‐NP loaded with scrambled siRNA. Further WB analysis of CD8^+^ T cells treated by supernatant showed that AS‐F‐NP treatment restored the levels of H3K79me2 and STAT5 in CD8^+^ T cells, providing a prerequisite for the secretion of IFN‐γ and maintenance of effector function (Figure 4I–H). Therefore, mechanistically, AS‐F‐NP can reduce the consumption and deprivation of methionine by knocking down Lat4 on tumor cells, ensuring that CD8^+^ T cells receive enough methionine for H3K79 di‐methylation to guarantee the expression of STAT5 and the production of IFN‐γ.

The greedy uptake of methionine also provides tumor cells with an ample feedstock pool of epigenetic modification. As a result, flexible plasticity of methylation ensures the malignant evolution of tumors and resistance to chemotherapy. For instance, trimethylation of H3K4 is the foundation for maintaining the phenotype of cancer stem cell subpopulations (CSCs). Therefore, given the inhibitory effect of AS‐F‐NP on methionine uptake by tumor cells, we further examined the changes in H3K4me3 and the CSC marker SOX‐9 and ALDH1 in 4T1 cells after AS‐F‐NP treatment. The results showed a significant reduction in the levels of H3K4me3, SOX‐9 and ALDH1 by AS‐F‐NP, with a stronger effect compared to AS‐NP without fluorination modification, indicating the enhance of fluorination modification on transfection efficiency (Figure 4L–N and Figure S22, Supporting Information). Thus, for tumor cells, AS‐F‐NP can reduce CSCs through the intervention in methionine uptake and epigenetic regulation, thereby alleviating chemotherapy resistance issues.

### 2.5 In Vivo Antitumor Efficacy of AS‐F‐NP

**Figure 5:**
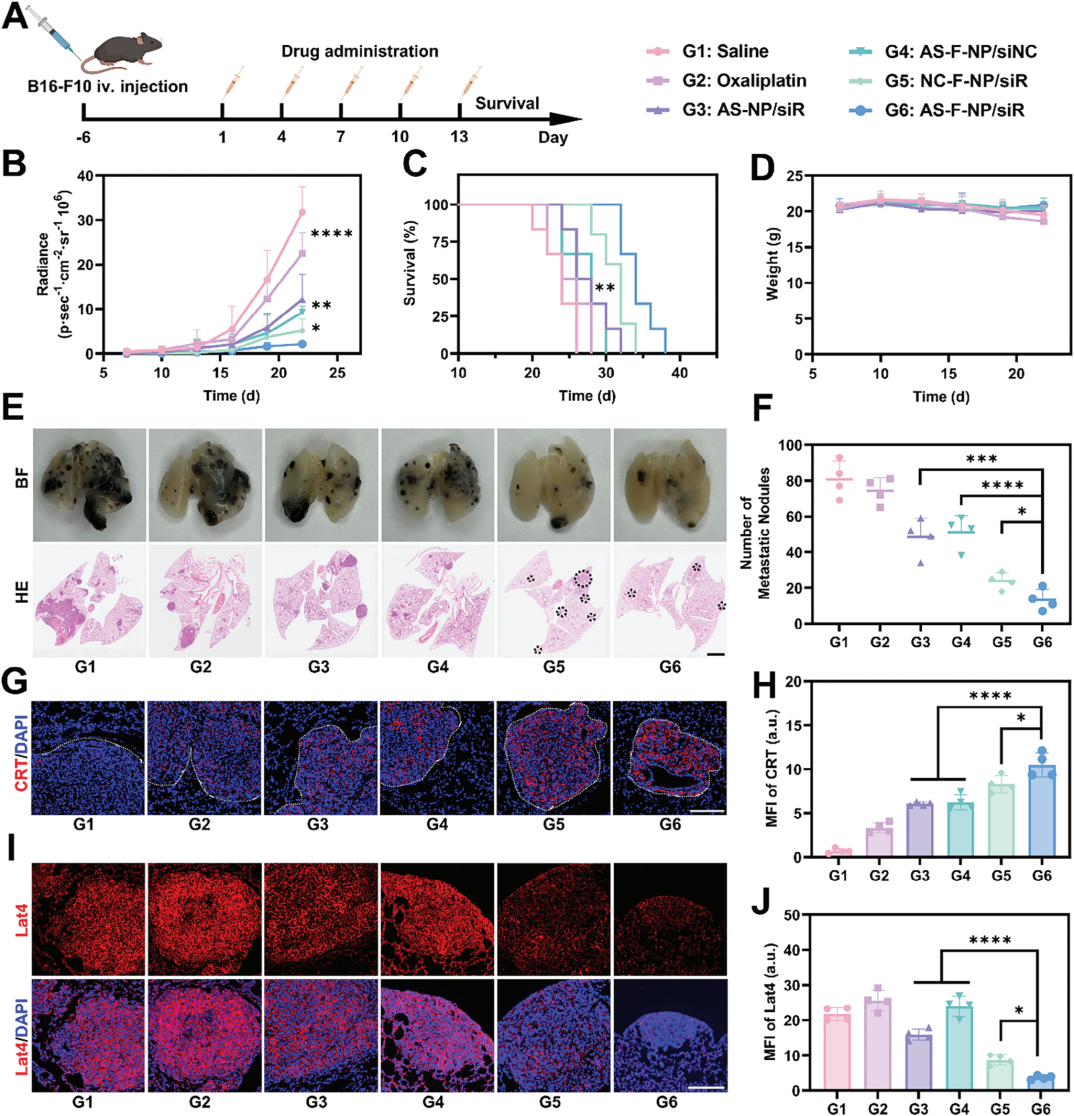
In vivo antitumor effect of AS‐F‐NP. A) Treatment plan and experiment grouping. B) Tumor growth curves, C) survival curves, and D) weight curves of B16‐F10 lung metastatic tumor bearing mice after different treatments. E) H&E staining of lungs in B16‐F10 bearing mice after different treatments (scale bar = 2 mm). F) Quantification results of lung metastatic nodules in B16‐F10 bearing mice after different treatments. Data are presented as means ± SD (*n* = 4). G) Immunofluorescence imaging and H) quantification results of mean fluorescent intensity of the CRT exposure in B16‐F10 lung metastatic nodules in mice with different treatment (scale bar = 200 µm). Data are presented as means ± S.D (*n* = 4). I) Immunofluorescence imaging and J) quantification results of mean fluorescent intensity of Lat4 in B16‐F10 lung metastatic nodules in mice with different treatment (scale bar = 200 µm). Data are presented as means ± SD (*n* = 4, one‐way ANOVA, Tukey's multiple comparisons test, *p < 0.1, **p < 0.01, ***p < 0.001, ****p < 0.0001).

Encouraged by the intense ICD effect and the epigenetic intervention based on methionine metabolism induced by AS‐F‐NP in vitro, in vivo experiments on the antitumor efficacy of different formulations were conducted on B16‐F10 melanoma lung metastasis‐bearing C57 mice. Treatment was initiated on the 6th day post‐B16‐F10 *iv*. injection modeling and administered five times, with tumor size reflected by monitoring bioluminescence signals using IVIS (**Figure** **5**A). As shown in Figure 5B,C, mice treated with AS‐F‐NP exhibited the lowest tumor signal and longest survival. Apart from a slight decrease in body weight observed in mice treated with saline and free OXA, possibly related to tumor burden and toxic side effects of free drugs, no other groups showed weight loss (Figure 5D). Additionally, based on the enumeration of melanoma metastatic nodules in the lungs of mice on the 25th day and hematoxylin‐eosin (HE) staining of lung tissues, mice treated with AS‐F‐NP exhibited the least number and smallest area of lung metastatic lesions (Figure 5E,F). These experimental results demonstrate the significant antitumor therapeutic effect of AS‐F‐NP in B16‐F10 lung metastasis‐bearing mice.

Furthermore, through immunofluorescence staining, it was found that AS‐F‐NP induced higher levels of CRT at the metastatic nodes compared to free OXA and AS‐NP without fluorination, indicating that the design incorporating the oxidized platinum (IV) and fluorination significantly enhanced ICD induction in vivo (Figure 5G,H). Correspondingly, the lungs of mice treated with AS‐F‐NP showed the highest recruitment of CD8^+^ T cells and TUNEL apoptosis signals in metastatic nodules (Figures S23 and S24, Supporting Information). Subsequent immunofluorescence staining of Lat4 revealed that the levels of Lat4 at the tumor nodes were significantly higher than in adjacent tissues, as expected (Figure 5I). Unexpectedly, it was found that the level of Lat4 in tumor tissues further increased after free OXA treatment, which may be related to acquired tumor resistance. Importantly, the administration of formulations containing siLat4 could reduce the expression of Lat4 at the tumor nodes, among which the fluorination modification significantly enhanced the gene silencing efficiency (Figure 5I,J). In addition, through quantitative analysis by LC‐MS/MS, we found that the methionine content in the lung tissues of the tumor‐bearing mice in various groups did not differ significantly, but the downstream metabolite S‐adenosyl‐methionine (SAM) and the ratio of SAM/S‐adenosyl‐homocysteine (SAH), which represents methylation availability (methylation index), were significantly reduced after treatment with the siLat4‐containing preparation (Figure S25, Supporting Information). This indicated that AS‐F‐NP achieved effective inhibition of tumor methionine consumption in vivo. Collectively, these results indicate that AS‐F‐NP effectively induces intense ICD in vivo, recruits CD8^+^ T cells to tumor nodes, and efficiently silences the upregulated Lat4 at the tumor nodes, demonstrating the potential of AS‐F‐NP to modulate immune response and overcome drug resistance by epigenetic intervene based on methionine restriction.

### 2.6 Intact Immune Response Feedback Loop

**Figure 6:**
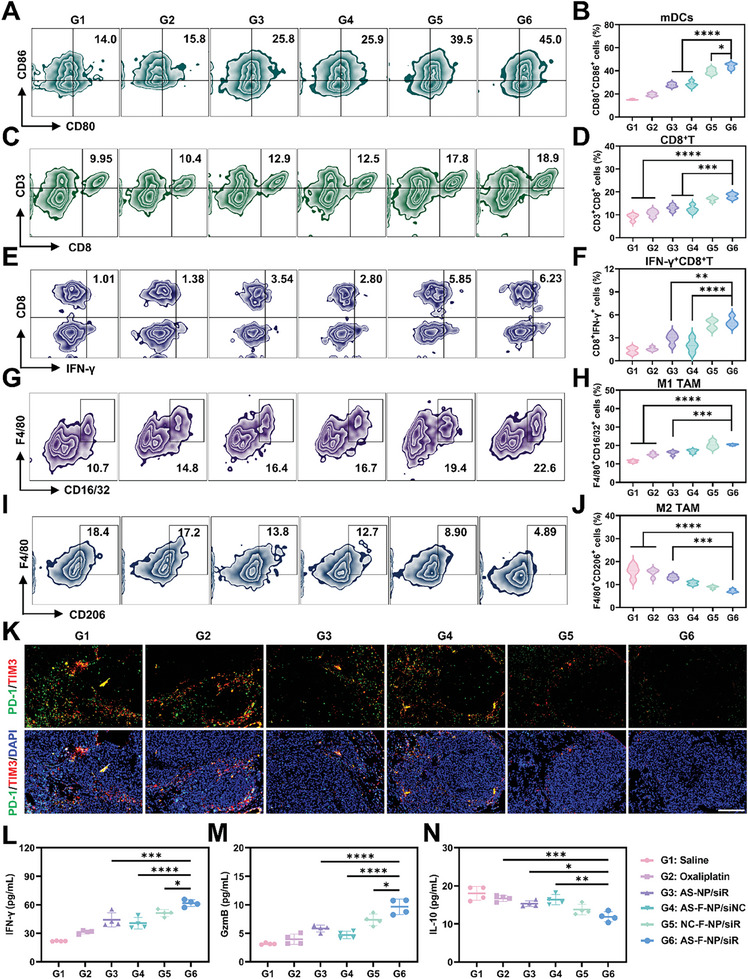
In vivo immunomodulation effect of AS‐F‐NP. A) FACS analysis and B) quantification results of CD80+ CD86+ DCs gating on CD11c+ cells within lymph. C) FACS analysis and D) quantification results of CD8+ T cells gating on CD45+ cells within lung. E) FACS analysis and F) quantification results of IFN‐γ+ CD8+ T cells gating on CD45+ CD3+ cells within lung. G) FACS analysis and H) quantification results of F4/80+ CD16/32+ TAMs gating on CD45+ cells within lung. I) FACS analysis and J) quantification results of F4/80+ CD206+ TAMs gating on CD45+ cells within lung. K) Immunofluorescence imaging of the infiltration of PD‐1+ TIM3+ T cells in B16‐F10 lung metastatic nodules in mice with different treatment (scale bar = 200 µm). (l – *n*) ELISA assay of L) IFN‐γ, M) GzmB, and N) IL‐10 in lungs of mice with different treatment. Data are presented as means ± SD (*n* = 4, one‐way ANOVA, Tukey's multiple comparisons test, *p < 0.1, **p < 0.01, ***p < 0.001, ****p < 0.0001).

Antitumor strategies based on immune rely on a complete immune positive feedback loop, as effective immune activation‐recruitment of immune cells‐tumor destruction by immune effector cells‐production of more tumor antigens to stimulate immune activation further. The ability of AS‐F‐NP to induce ICD and recruit T cells at the in vivo level prompted us to further comprehensively investigate the overall tumor immune microenvironment to evaluate the contribution of AS‐F‐NP to the immune response positive feedback loop. After the fifth administration, the immune activation status in the lung metastatic tumor tissue and draining lymph nodes of the mice was examined by FACS. Mice treated with AS‐F‐NP had the highest proportion of mature DCs in the draining lymph nodes and the most CD8^+^ T cell recruitment in lungs, indicating a strong immune activation induced by ICD (**Figure** **6**A–D). Moreover, based on the in vitro validation experiments of methionine balance intervention and epigenetic regulation, the function and phenotype of immune cells in vivo were further assessed to reflect the global changes in the tumor immune microenvironment after AS‐F‐NP treatment. The results demonstrated the crucial role of methionine metabolism intervention by AS‐F‐NP in the effector functionality of CD8^+^ T cells infiltrating the tumor. With the addition of fluorination and aptamer modification, AS‐F‐NP could mediate the highest proportion of IFN‐γ^+^CD8^+^ T cells in the tumor (Figure 6E,F). Consequently, AS‐F‐NP treatment led to changes in the overall immune microenvironment of the tumor site, including the transformation of protumor M2‐type tumor‐associated macrophages (TAMs) into antitumor M1‐type TAMs and a reduction in the proportion of exhausted T cells (Figure 6G–K). Finally, ELISA analysis showed changes in the levels of immune‐related cytokines in the lungs of mice treated by different formulations (Figure 6L–N). AS‐F‐NP treatment induced the highest levels of IFN‐γ and Granzyme B (GzmB), closely related to the effector function of CD8^+^ T cells, and the lowest levels of the immunosuppressive cytokine IL‐10. These results indicate that AS‐F‐NP not only initiates immune activation response programs resulted from inducing intense ICD but also influences the function and phenotype of tumor infiltrating immune cells and the levels of cytokines in the overall immune microenvironment through methionine distribution intervention and epigenetic modification, forming a closed immune positive feedback loop ultimately mediating a powerful antitumor immune effect.

### 2.7 Elimination of CSCs and Chemotherapy Sensitization

**Figure 7:**
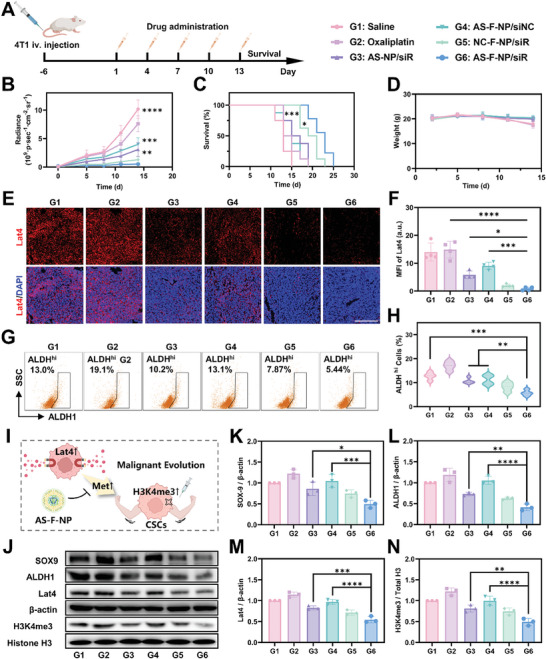
In vivo CSC suppressive effect of AS‐F‐NP. A) Treatment plan and experiment grouping. B) Tumor growth curves, C) survival curves, and D) weight curves of 4T1 lung metastatic tumor bearing mice after different treatments. E) Immunofluorescence imaging and F) quantification results of mean fluorescent intensity of Lat4 in B16‐F10 lung metastatic nodules in mice with different treatment (scale bar = 200 µm). Data are presented as means ± SD (*n* = 4). G) FACS analysis and (H) quantification results of ALDHhi cells in lungs of 4T1 lung metastatic tumor bearing mice with different treatment. I) Schematic illustration of treatment mechanism. J) Western blot assay and semiquantification analysis of K) SOX‐9, L) ALDH1, M) Lat4, and N) H3K4me3 in lungs of 4T1 lung metastatic tumor bearing mice with different treatment. Data are presented as means ± SD (*n* = 3, one‐way ANOVA, Tukey's multiple comparisons test, *p < 0.1, **p < 0.01, ***p < 0.001, ****p < 0.0001).

To validate the efficacy of AS‐F‐NP against another malignant lung metastatic tumor, a 4T1 lung metastatic tumor model in BALB/c mice was also established and treated with different formulations, obtaining similar therapeutic results as described above (**Figure** **7**A–D and S26, Supporting Information). Through immunofluorescence staining of lung metastatic tumor sections, it was found that free OXA treatment alone increased the expression level of Lat4, while decreased expression in AS‐F‐NP treatment group, as the same as former results. Given the crucial contribution of Met to H3K4 trimethylation, we speculated that free OXA treatment would induce tumor cells to alter their metabolic patterns further to replenish the methyl donor pool to ensure their sufficient H3K4me3 and drive the evolution of the chemotherapy‐resistant CSCs phenotype. Therefore, the changes in the proportion of CSCs (ALDH^hi^ cells) at the tumor site were examined through FACS (Figure 7G,H). As expected, the proportion of ALDH^hi^ cells significantly increased after free OXA treatment, contrasting the results of AS‐F‐NP treatment. Additionally, the proportion of CSCs did not decrease after treatment with formulations containing scrambled siRNA, indicating the importance of restricting methionine uptake and its downstream epigenetic modification in inhibiting CSCs (Figure 7I). Further support for these results was obtained through WB analysis (Figure 7J–N). In conclusion, the tumor suppressive efficacy of AS‐F‐NP has been validated on different tumors derived from two different strains of mice and this co‐delivery system can efficiently eliminate CSCs through methionine uptake intervention and epigenetic modification to sensitize tumors to chemotherapy.

### 2.8 Biocompatibility and Safety of AS‐F‐NP In Vivo

Lastly, we conducted a comprehensive evaluation of the biocompatibility and safety of AS‐F‐NP. After treatment of C57 mice bearing tumor, plasma levels of liver and kidney function‐related markers were measured, all falling within the safe range values, indicating that AS‐F‐NP does not cause additional undesirable toxicity of major metabolic and excretory organs (Figure S27, Supporting Information). Additionally, HE staining of major organs showed that besides the spleen abnormalities associated with hypersensitivity reactions to free OXA, treatments with other formulations did not result in significant organ damage (Figure S28, Supporting Information). Similar results were verified in 4T1‐bearing BALB/c mice (Figure S29, Supporting Information). These results demonstrate the satisfactory biocompatibility and in vivo safety of AS‐F‐NP, highlighting its clinical translational potential.

## 3 Discussion

Epigenetic dysregulation is a prevalent feature of malignant tumor progression. Adaptive metabolic patterns not only serve as the underlying conditions for epigenetic dysregulation but also directly propel tumor cell evolution and stress the tumor microenvironment. The addictive uptake and dependent demand for methionine by tumor cells have been well‐documented. Recent studies have unveiled that methionine deprivation behavior also plays a crucial role in coercing immune cells toward an inhibitory phenotype, fostering a procancer immune microenvironment.

This study aims to develop a co‐delivery therapeutic platform that integrates methionine metabolic modulation strategies with chemotherapy. By targeting the Lat4 transporter, which influences methionine distribution imbalance in the tumor microenvironment, we devised a combined therapeutic system for co‐delivery of siRNA and OXA. Enhancements in stability, transfection efficiency, and tumor targeting of this co‐delivery system were achieved through fluorination and aptamer modification. The strategy of oxidized OXA cross‐linking not only augmented the efficiency of immunogenic cell death (ICD) induction but also conferred tumor‐responsive drug‐release properties to the formulation. This resultant co‐delivery system demonstrated robust ICD induction both in vitro and in vivo, reinstating a positive feedback loop of the immune response and inhibiting cancer stem cells, by intervene methionine distribution imbalance, leading to superior antitumor efficacy in two distinct tumor models. Moreover, this co‐delivery system exhibited a favorable in vivo safety profile and could potentially serve as a versatile platform for delivering arbitrary sequence siRNAs for antitumor therapy.

## 4 Experimental Section

### Chemicals and Materials

2,2,3,3,4,4,5,5,6,6,7,7,7‐Tridecafluoroheptyloxirane (>98.0% (GC)) was purchased from Aladdin (Shanghai, China). Polyethyleneimine (branched, M.W.1200, 99%) was purchased from Energy Chemical (Shanghai, China). OXA, D‐Luciferin potassium, Annexin V‐FITC/PI Apoptosis Detection Kit, Hoechst 33342 Staining Solution and LysoTrackerGreenDND‐26 were purchased from Meilunbio (Dalian, China). Methoxy PEG Succinimidyl Carboxymethyl Ester (mPEG‐NHS, Mw = 3400) and Maleimide PEG Succinimidyl Carboxymethyl Ester (mal‐PEG‐NHS, Mw = 3500) were purchased from Jenkem technology (Beijing, China). Sodium L‐Ascorbate (99%) was purchased from Adamas (Shanghai, China). l‐Methionine was purchased from Macklin (Shanghai, China). RPMI‐1640 (Dutch modified) (methionine‐free, HEPES) was purchased from Procell (Wuhan, China). Gel‐Red, Dihydroethidium (DHE), Histone H3 Mouse Monoclonal Antibody, β‐Actin Mouse Monoclonal Antibody and Antifade Mounting Medium with DAPI were purchased from Beyotime (Shanghai, China). Anti‐HMGB1 antibody (ab18256) and anticalreticulin antibody (ab92516) were purchased from Abcam (Cambridge, MA, UK). Anti‐SLC43A2 pAb (abs100575) was purchased from Absin (Shanghai, China). STAT5 Rabbit mAb (A5029), DiMethyl‐Histone H3‐K79 Rabbit pAb (A2368), SOX9 Rabbit mAb (A19710), TriMethyl‐Histone H3‐K4 Rabbit pAb (A2357), ALDH1A1 Rabbit mAb (A0157) and ELISA assay kits (IFN‐γ, Gzmb and IL‐10) were purchased from Abclonal (Shanghai, China).

siLat4 (S: AGCUGUUCCUUAAGAUCAATT; AS: UUGAUCUUAAGGAACAGCUTT), Cy5‐siLat4, siNC (S: UUCUCCGAACGUGUCACGUTT; AS: ACGUGACACGUUCGGAGAATT) and Cy5‐siNC were purchased from GenePharma (Shanghai, China). AS1411‐SH (Sequence (5′ to 3′): GGTGGTGGTGGTTGTGGTGGTGGTGG), FAM‐AS1411‐SH, NC‐Apt‐SH (Sequence (5′ to 3′): GCGTAGTGATTATGAATCGTGTGCTA) and FAM‐NC‐Apt‐SH DNA aptamers were purchased from Sangon Biotech (Shanghai, China).

### Cells

4T1 cells, luciferase‐expressing 4T1 (4T1‐Luc) cells, ERFP‐expressing 4T1 (4T1‐ERFP) cells, B16‐F10 and luciferase‐expressing B16‐F10 (B16‐F10‐Luc) cells were purchased from Stem Cell Bank, Chinese Academy of Sciences (Shanghai, China). All the cells were cultured in RPMI‐1640 medium (Gibco, Carlsbad, CA, USA) supplemented with 10% fetal bovine serum (FBS) (Gibco, Carlsbad, CA, USA) and 1% antibiotics (penicillin and streptomycin) at 37 °C under the air atmosphere of 5% CO_2_.

### Animals

C57BL/6 male mice (6–8 weeks, 18–22 g, SPF) and Balb/c female mice (6–8 weeks, 18–22 g, SPF) were purchased from Slac Laboratory Animal Co. Ltd. (Shanghai, China). All of our animal experiments were managed in conformity with guidelines evaluated and approved by Fudan University Institutional Animal Care and Use Committee (2020‐04‐YJ‐ST‐01). As to the noninvasive monitoring of the tumor growth, the tumor‐bearing mice were administrated with 150 µL d‐luciferin potassium solution (20 mg mL^−1^, MeilunBio, Dalian, China) by intraperitoneal injection 10 min before the IVIS observation (PerkinElmer Inc., Waltham, MA, USA). The mice were monitored for clinical signs every two days to assess behavioral and locomotor parameters, including activity levels, posture, piloerection, self‐mutilation, and food and water intake. These observations were used to evaluate the progression of tumor burden and to monitor the ethical limits of tumor growth. Humane endpoints for euthanasia were established and included the presence of lethargy, weakness, a hunched posture, signs of pain, reduced ability to access food and water, rapid, or labored breathing, neurological symptoms (such as a tilted head, uneven gait, or paralysis), or a body weight loss exceeding 20%. Euthanasia was performed using isoflurane anesthesia.^[^ ^23^, ^24^ ^]^

### Samples of Patient Sources

The primary tumor tissues and peritumor tissues of breast cancer patients were obtained from Ruijin Hospital, Shanghai Jiaotong University School of Medicine (Shanghai, China). The examinations were approved by the Ruijin Hospital Ethics Committee (AF0406/14.0/2023‐03‐01 or (2024) Provisional Ethics Approval No.15) and all patients provided informed consent.

### Gene Expression Profiling Interactive Analysis (GEPIA)

GEPIA (http://gepia2.cancer‐pku.cn) is an innovative internet platform providing customized analyses of gene expression in multiple cancers, including differential expression analysis, correlation analysis, and survival analysis, using the RNA sequencing data of 9736 tumors and 8587 normal samples from TCGA databases and Genotype‐issue Expression (GTEx) projects.^[^ ^25^ ^]^

### Synthesis of PEG‐Pt‐F‐PEI and PEG‐Pt‐PEI

A total of 100 mg oxaliplatin and 20 mL of H_2_O_2_ (30%) were added to a 50 mL round bottom flask and stirred at room temperature for 12 h. The round bottom flask was stirred open and heated to 50 °C for 30 min. The reaction solution was freeze‐dried to give Compound **2**.

A total of 100 mg of compound **2** and 232 mg of succinic anhydride were added to a 100 mL bicarbonate flask containing 40 mL of dry DMF, which was heated to 70 °C and stirred under argon protection for 24 h. The reaction solution was concentrated and added dropwise to dichloromethane (v:v = 1:25) and then centrifuged at 8000 rpm for 5 min before discarding the supernatant. The precipitate was dried under vacuum and compound **3** was obtained.

A total of 1 g of polyethyleneimine (PEI_1.2k_), 627 mg of compound **5**, and 100 mL of methanol were added to a 250 mL round‐bottomed flask and the reaction was heated to 60 °C and stirred for 15 h. The reaction solution was loaded into a 1 kDa dialysis bag and dialyzed in 3 L of methanol for 24 h before being distilled under reduced pressure to a viscous consistency, at which point compound **6** was obtained.

A total of 500 mg of compound **3**, 430 mg of N‐hydroxythiosuccinimide, 380 mg of 1‐ethyl‐3 (3‐dimethylpropylamine) carbodiimide, and 6 mL of 0.05 M PBS (ph = 6.0) were added to a 25 mL single‐necked vial and activated with stirring at room temperature for 1 h. Subsequently 515.6 mg of compound **6** and 6 mL of 0.1 M sodium carbonate buffer (ph = 8.3) were rapidly added to the vial and the reaction was continued with stirring for 24 h. The reaction solution was dialyzed in a 10 kDa dialysis bag in 5 L of deionized water for 24 h. The water was changed every 6 h. The dialyzed reaction solution was filtered through a 0.45 µm filter membrane and freeze‐dried, and compound **7** was obtained.

A total of 500 mg of compound **7**, 77 mg of compound **8,** and 20 mL of 0.05 M PBS (ph = 7.2) were added to a 50 mL single‐necked vial and reacted with stirring at room temperature for 5 h. The reaction solution was purified by ultrafiltration for three times (10 kDa, 10000 g), and the upper layer of the liquid was freeze‐dried to give compound **9**.

Compound **12** was synthesized by a similar method as described above.

### Preparation of F‐NP and AS‐F‐NP Suspensions

PEG‐Pt‐F‐PEI was dissolved in DEPC water at a gradient concentration, mixed with an equal volume of siRNA solution (20 µM), vortexed for 30 s and then allowed to stand for 30 min to obtain F‐NP suspensions with different N/Ps. The optimal N/P was determined by agarose gel electrophoresis and DLS assay. F‐NP was mixed with AS1411‐SH (10 µM, v/v = 2/1), mixed by gentle blowing and left to stand for 1 h to obtain AS‐F‐NP suspensions, and the particle size distribution and potentials were determined by DLS. In addition, dual fluorescence‐labeled AS‐F‐NP suspensions were prepared with Cy5‐siRNA and FAM‐AS1411‐SH, which were cured by Matrix‐gel (Beyotime, Shanghai, China) and observed under CLSM. The fluorine‐free control nanoparticle (NP) suspensions were obtained with the same N/P and similarly prepared.

### Agarose Gel Electrophoresis

The 3% agarose gel with Gel‐Red (1:10 000) was prepared for electrophoresis of F‐NP suspensions at different N/P, with free siRNA as a negative control and the gold standard PEI_25k_ as a positive control (siRNA:100 ng well^−1^). Electrophoresis was carried out for 15 min at a constant pressure of 100 V followed by development imaging.

The methods in the encapsulation effeciency (EE) determination was similar to that described above. AS‐F‐NP/siRNA with or without excess sodium heparin (2 mg mL^−1^) and gradient concentrations of siRNA (16.5, 33.0, 66.0, 132, 264 ng/µL) were subjected to agarose gel electrophoresis simultaneously. The encapsulation effeciency (EE) was calculated by quantifying the light intensity in the imaging results.

(1) $$\mathrm{Encapsulation}\;\mathrm{Effieciency}\;\left(\mathrm{EE}\right)=\frac{\mathrm{Total}\;\mathrm{siRNA}\;\mathrm{mass}-\mathrm{free}\;\mathrm{siRNA}\;\mathrm{mass}}{\mathrm{Total}\;\mathrm{siRNA}\;\mathrm{mass}}$$

The 3% agarose gel without Gel‐Red was prepared for electrophoretic examination of FAM‐AS1411‐SH covalent coupling, with a physical mixture of F‐NP and FAM‐AS1411 as a control (AS1411:100 ng well^−1^). fluorescence development imaging was performed after electrophoresis at a constant pressure of 100 V for 15 min (channel: 488/520).

### Transmission Electron Microscopy (TEM)

The prepared nanoparticle suspension was diluted to a suitable concentration and added dropwise onto the hydrophilic pretreated support membrane for 1 min of adsorption, and then the excess liquid was removed by blotting paper and the support membrane was dried. Then 2% phosphotungstic acid solution was added dropwise for 1 min of staining, and the excess liquid was removed by blotting and air‐drying before imaging.

### Drug Release Evaluation

Leakage of siRNA from nanoparticles was examined by agarose gel electrophoresis after incubating AS‐F‐NP suspensions with PBS 7.4 or PBS 7.4 (10 mM VcNa) that mimics the reducing environment in tumor cells at 37 °C for different times. Competitive replacement of all siRNAs with the addition of heparin was used as a positive control.

Changes in the particle size distribution of the nanoparticles were examined by DLS at the 12th h of incubation. Changes in nanoparticle morphology after incubation with VcNa‐containing PBS were observed by TEM.

The responsive release of OXA from the prodrug polymer was evaluated by incubation with PBS 7.4 or PBS 7.4 (10 mM VcNa) at 37 °C and quantified by HPLC with samples taken at different time points (HPLC assay method: column = Agilent C18 250 mm × 4.6 mm, mobile phase = 90% methanol–10% water, flow rate = 1 mL min^−1^, injection volume = 20 µL, DAD = 250 nm).

### Cellular Uptake and Intracellular Distribution

B16‐F10 cells or 4T1 cells were inoculated into 24‐well plate (3 × 10^4^ cells well^−1^) and cultured for 24 h. Then, the medium was removed and the cells were washed with HBSS for three times. The cells were incubated with serum‐free RPMI 1640 medium containing different nanoparticles double‐labeled with Cy5 (siRNA, 300 nM) and FAM (Aptamer, 150 nM) at 37 °C for 0.5 h. After washing with HBSS, the cells were observed under inverted fluorescence microscope (Olympus IX73, Olympus, Japan) or collected and the fluorescence intensity was detected with flow cytometry (Beckman, Indianapolis, USA).

For an examination of the mechanism of uptake, B16‐F10 cells were pretreated with different uptake inhibitors at 37 °C for 0.5 h, including filipin (5 µg mL^−1^), chlorpromazine (5 µg mL^−1^), and colchicine (10 µg mL^−1^), to inhibit caveolin‐mediated endocytosis, clathrin‐mediated endocytosis, and macropinocytosis, respectively. Following three washes with HBSS, the cells were incubated with AS‐F‐NP (Cy5‐siRNA, 300 nM) at 37 °C for 0.5 h. An additional group of cells was cultured at 4 °C for 0.5 h to examine the energy‐dependency of nanoparticle internalization. After washing and harvesting, fluorescence intensity (channel: APC) was measured by using a flow cytometer (Beckman, Indianapolis, USA).

For the examination of intracellular processes, B16‐F10 cells were cultured in a confocal dish at a density of 1 × 10^4^ cells well^−1^ for 24 h. Cy5‐labeled AS‐F‐NP were then added and incubated for either 0.5, 1, 3, and 6 h. After washing with HBSS, the cells were treated with LysoTracker Green DND‐99 (50 nM) and Hoechst 33342 (5 µg mL^−1^) for 15 min at 37 °C. Following other washes with HBSS, CLSM was used to visualize the localization of AS‐F‐NP and lysosomes (SpinSR10, Olympus, Japan). As for the time‐lapse imaging, cells were first co‐incubated with LysoTracker Green DND‐99 (50 nM) and Hoechst 33342 (5 µg mL^−1^) for 15 min at 37 °C. Cy5‐labeled AS‐F‐NP was then added and imaging was initiated every 3 min for successive 1 or 6 h periods.

### In Vivo Clearance of siRNA

Healthy C57BL/6 mice were divided into two groups, and after being injected with free Cy5‐siRNA (1 mg kg^−1^) or AS‐F‐NP suspension containing an equal dose of Cy5‐siRNA by tail vein, blood was taken at 0.5, 1, 2, 4, 8, 12, and 24 h. The fluorescence intensity was measured by IVIS Spectrum Imaging System (PerkinElmer Inc., Waltham, MA, USA) to determine the fluorescence intensity.

### In Vivo Tumor Targeting and Biodistribution

The 4T1‐ERFP cells were injected into BALB/c mice at 5 × 10^5^ cells/mouse via tail vein to prepare a lung metastasis cancer model. The mice were imaged under the IVIS Spectrum imaging system (PerkinElmer) to determine the formation of lung metastases. Six days post of the injection, Cy5 labeled NC‐F‐NP and AS‐F‐NP suspensions were respectively injected into the lung metastasis model at 0.5 mg kg^−1^ of Cy5 via the tail vein injection. The in vivo distribution at different time points was tracked by the IVIS system. 24 h after the injection, the mice were sacrificed and the major organs (heart, liver, spleen, lung, kidney) were collected and monitored with IVIS system. Then the lungs were fixed in 4% paraformaldehyde. The lung sections at 8 µm were obtained after embedded in optimal cutting temperature (OCT) compound and were visualized under the confocal fluorescence microscope (CLSM, SpinSR10, Olympus, Japan).

By the same method as described above, lungs from B16‐F10 lung metastasis models of C57BL/6 mice were obtained and fixed, subsequently embedded in OCT and prepared as 8 µm sections before visualization under the CLSM.

### Intracellular ROS Assay

Dihydroethidium (DHE) was employed to assess the intracellular ROS in vitro. DHE, a commercially available probe, detects superoxide anions and exhibits a response to other types of ROS, such as H_2_O_2_ and ^•^OH. Briefly, B16‐F10 cells or 4T1 cells were plated in a 12‐well plate (1 × 10^5^ cells well^−1^) and cultured for 24 h before use. After different treatment for 24 h, cells were washed with HBSS before incubated with medium containing DHE (2 µM) for 0.5 h. Then, the cells were observed with an inverted fluorescence microscope (Olympus IX73, Olympus, Japan) or collected for a FACS analysis.

### Evaluation of ICD Inducing

For the detection of CRT on the cell surface, B16‐F10 cells or 4T1 cells were inoculated to 4‐chamber glass bottom dishes (1 × 10^4^ cells well^−1^) for 12 h. After treated with different formulations for 24 h, cells were washed with HBSS and fixed in 0.1% paraformaldehyde in cold HBSS for 5 min. Then, the cells were washed again, and the Alexa Fluor 488 conjugated CRT antibody diluted in cold blocking buffer was added for 1 h at 4 °C. After washes in cold HBSS, the cells were incubated with WGA‐630 (2 µg mL^−1^) and Hoechst‐33342 (5 µg mL^−1^) in HBSS for 15 min. In the end, the cells were visualized by CLSM. As for FACS analysis, the cells were inoculated to 12‐well plate (1 × 10^5^ cells well^−1^) and then were treated with different formulations for 24 h. After washes in cold HBSS, the cells were collected and blocked in cold HBSS with 5% BSA for 10 min. Then, the cells were incubated with the Alexa Fluor 488 conjugated CRT antibody at 4 °C for 1 h. After washes in cold HBSS, the cells were prepared for FACS analysis (channel: FITC).

For the detection of HMGB1 release, the cells were inoculated to 4‐chamber glass bottom dishes (1 × 10^4^ cells well^−1^) for 12 h. After treated with the same procedure mentioned in CRT detection, the cells were rinsed three times with cold HBSS, fixed in 4% paraformaldehyde for 10 min, permeabilized with 0.1% Triton X‐100 for 10 min and washed thrice with cold HBSS. After blocked in cold HBSS with 5% BSA for 10 min, the cells were incubated with HMGB1 primary antibody at 4 °C for 12 h. Subsequently, the cells were washed incubated with an Alexa Fluor 488‐conjugated secondary antibody for 2 h, and stained with Hoechst‐33342 (5 µg mL^−1^) for 15 min. The HMGB1 release from nucleus was visualized by CLSM.

In addition, B16‐F10 cells were cultured in 12‐well plates at 8 × 10^5^ cells well^−1^ for 12 h, and then AS‐F‐NP with different Pt concentrations (10, 20, and 40 µM) was administered for 24 h, and the supernatants were centrifuged at 3000 rpm for 10 min. The supernatants were concentrated in 10 kDa ultrafiltration tubes, and then the HMGB1 content of supernatants was determined by Western blot.

For the mature of dendritic cells (DCs), the immature DCs were obtained following the previous literature, which were inoculated into 6‐well plate (4 × 10^5^ cells well^−1^) for 24 h.^[^ ^26^ ^]^ Then, the DCs were treated with different supernatants of B16‐F10 tumor cells after incubated with different formulations for 24 h. In the end, the DCs were collected to analyze the maturation by flow cytometry (Alexa Fluor 488‐conjugated CD11c antibody, PE‐conjugated CD86 antibody and APC‐conjugated CD80 antibody).

### Western Blot

For the Western blot assay, the B16‐F10 cells or 4T1 cells were inoculated in 6‐well plate (1 × 10^5^ cells well^−1^) for 12 h, then were treated with different formulations (siRNA = 100 nM) for 48 h in serum‐free RPMI 1640 with 30 µM methionine.

The cell samples or freshly excised lung metastatic tumors were lysed with RIPA lysis buffer containing protease inhibitor cocktail. The protein concentration of the cell sample was measured by BCA Protein Assay kit (Beyotime Biotechnology, Shanghai, China). For cell samples, total proteins (20 µg/hole) were separated by 4–20% SDS‐PAGE electrophoresis at 120 V for 2 h. For tissue samples, the concentration of the total protein was 40µg/hole. Afterward, the protein was transferred to PVDF membranes, which were blocked with quick‐block buffer (Beyotime Biotechnology, Shanghai, China) for over 15 min. The incubation of the primary antibody (Lat4, Absin, 1:1000; STAT5, Abclonal, 1:1000; β‐actin, Beyotime, 1:1000; H3K79me2, Abclonal, 1:1000; Histone H3, Beyotime, 1:1000; SOX‐9, Abclonal, 1:1000; H3K4me3, Abclonal, 1:1000; ALDH1A1, Abclonal, 1:1000) lasted overnight. After washed with TBST buffer three times for 10 min, the membranes were incubated with antirabbit or antimouse secondary antibodies (1:1000) conjugated with horseradish peroxidase (HRP) for 1 h. Then the membranes were washed three times for 10 min with TBST buffer. The protein expression levels were detected by enhanced chemiluminescence autoradiography with the use of using ECL plus. All the gray statistics diagrams of WB in the manuscript were analyzed by ImageJ.

### Methionine Consumption Assay

B16‐F10 cells or 4T1 cells were inoculated into 6‐well plate (1 × 10^5^ cells well^−1^) for 12 h. Then the cells were treated with different formulations (siRNA = 100 nM) for 48 h in serum‐free RPMI 1640 with 30 µM methionine, which simulates the physiological concentration. In the end, the cell supernatants were collected and centrifuged at 3000 rpm for 5 min, and then three times volume of protein precipitant (acetonitrile: water: acetic acid = 100:10:0.1) was added to the supernatants. After vortex, the supernatants were centrifuged at 15 000 × *g* for 10 min, then was evaporated dry, concentrated and reconstituted with the mobile phase for quantitative determination by HPLC.

As for in vivo methionine sonsumption assays, 0.02 g of tumor‐containing lung tissue in 0.2 mL of water was thoroughly ground before 0.6 mL of protein precipitant (methanol containing 1% acetic acid) was added. After thorough mixing for 30 s, the suspension was centrifuged at 13 000 rpm for 10 min at 4 °C, and the supernatant was collected for quantitative LC–MS/MS analysis.

### LC–MS/MS Conditions

The LC–MS/MS analysis was performed using a AB Sciex 4000 QTRAP LC/MS/MS (AB Sciex Pte. Ltd., Netherlands) equipped with an LC‐20AD system (Shimadzu, Kyoto, Japan) and SIL‐20A autosampler (Shimadzu, Kyoto, Japan). Chromatographic separation was performed using gradient elution on the Agilent 5 TC‐C18(2) (250 × 4.6 mm, 5 µm) column (Agilent Technologies Santa Clara, CA, USA). The mobile phases were as follows: A, 10 mM ammonium acetate in water; and B, 20% (v/v) acetonitrile in methanol. The flow rate was 0.8 mL mi^−1^n. The gradient employed was as follows: 0.01–1.5 min started at 5% B, 1.5–6 min from 5 to 50% B, 6–7 min from 50 to 95% B and hold for 1 min, 8–9 min from 95 to 5% B and hold for 4 min. The total time was 13 min. The temperature of column oven was maintained at 40 °C, and the injection volume was 3 µL in each run.

The detection of the metabolites was carried out using positive electrospray ionization technique and selected reaction monitoring mode. The precursor → product transitions for Met (*m*/*z* 150.0 → 105.9), SAM (*m*/*z* 399.0 → 250.0) and SAH (*m*/*z* 385.1 → 136.2) were monitored. The MS operating conditions were as follows: entrance potential: 10.0 kV; collision cell exit potential: 15.0 kV; collision gas: medium; ion source gas 1: 50.0 psi; ion source gas 2: 50.0 psi.

### Evaluation of CD8 T Cell Viability and Function

The spleen of healthy C57 mice was taken to obtain primary T cells according to the kit method. The primary T cells were activated by CD3 antibody (2 µg mL^−1^) and CD28 antibody (2 µg mL^−1^) for 48 h, and were maintained with IL‐2 (50 ng mL^−1^) and 2‐mercaptoethanol (50 µM). Then, the T cells were seeded in a 6‐well plate (6 × 10^5^ cells well^−1^) with fresh medium, tumor supernatant supplemented with methionine or different tumor supernatants described above for 36 h. In the end, the T cells were collected to analyze the apoptosis and function by flow cytometry (PE‐conjugated CD8a antibody, FITC‐conjugated Annexin V and eFluor 450‐conjugated IFN‐gamma antibody).

### In Vivo Antitumor Efficacy

C57BL/6 mice were injected *iv*. with 5 × 10^5^ B16‐F10‐luci tumor cells to prepare the lung metastasis cancer model. 6 days after injection, the mice were divided into six groups (*n* = 10 each group) randomly according to the tumor size (the bioluminescent intensity of initial tumors: ≈2 × 10^5^ psec^−1^ cm^−2^ sr^−1^). Saline, OXA or different formulations were administered on day 6, 9, 12, 15, and 18, respectively. The formulations include G1: Saline (mice were administered an equivalent volume of saline via *iv*.); G2: OXA (mice received an injection of 5 mg kg^−1^ of free oxaliplatin via *iv*.); G3: AS‐NP/siR (mice were injected with an AS1411‐modified nanocomplex of oxaliplatin‐crosslinked unfluoridated polycation compressing siLat4 via *iv.)*; G4: AS‐F‐NP/siNC (mice were injected with an AS1411‐modified nanocomplex of oxaliplatin‐crosslinked fluoridated polycation compressing siNC via *iv*.); G5: NC‐F‐NP/siR (mice were injected with a scrambled‐aptamer modified nanocomplex of oxaliplatin‐crosslinked fluoridated polycation compressing siLat4 via *iv.)*; G6: AS‐F‐NP/siR(mice were injected with an AS1411‐modified nanocomplex of oxaliplatin‐crosslinked fluoridated polycation compressing siLat4 via *iv.)*. The dose of OXA is 5 mg kg^−1^. The dose of siRNA is 1 mg kg^−1^. The mice weight and survival data were respectively recorded. Four mice were randomly selected from each group and sacrificed at day 25 to count the surface metastatic nodules, and further prepare for immunofluorescence analysis and Western blot assay.

The lung metastasis models of 4T1‐luci‐bearing BALB/c mice were constructed and administrated follow the above methods (the bioluminescent intensity of initial tumors: ≈1 × 10^7^ psec^−1^ cm^−2^ sr^−1^). The mice were sacrificed at day 20 to prepare for immunofluorescence analysis, FACS, and Western blot assay.

### Analysis of Immune Cell Distribution and Activation in Tumor

Tumor draining lymph nodes (TDLN) and lungs with tumors of mice in different groups were harvested and dispersed into single‐cell suspensions by using 70 µm cell strainers (BD Pharmingen, New Jersey, America), respectively. Then, the TDLN cells and tumor‐infiltrating lymphocytes were quantitatively analyzed by flow cytometry following immunofluorescence staining. To investigate the immune cell infiltration and activation in the tumors and TDLNs, the single‐cell suspensions were stained with antibodies of well‐accepted biomarkers of immune cells according to the manufacturer's protocols. All the antibodies were presented as following: T cell (anti‐CD45‐APC, eBioscience, 17‐0451‐82; anti‐CD3‐FITC, 11‐0032‐82; anti‐CD8a‐PE, eBioscience, 12‐0081‐82; anti‐IFN‐γ‐eFluor 450, eBioscience, 48‐7311‐82); Macrophages (anti‐CD45‐APC, eBioscience, 17‐0451‐82; anti‐F4/80‐eFluor 450, eBioscience, 48‐4801‐82; anti‐CD16/32‐PE, eBioscience, 12‐0161‐82; anti‐CD206‐PE‐Cyanine7, eBioscience, 25‐2061‐82); DCs (anti‐CD11c‐PerCP‐Cyanine5.5, eBioscience, 45‐0114‐80; anti‐CD80‐Super Bright 436, eBioscience, 62‐0801‐82; anti‐CD86‐PE, eBioscience, Catalog12‐0862‐82). All ELISA results were obtained according to manufacturer's protocols of Abcam plc.

### Immunofluorescence Staining

The lungs with tumors were harvested and sliced into slides as described above, which were then immunofluorescence stained with anti‐Lat4 rabbit antibody (Absin, ABS100575), anticalreticulin antibody (Abcam, ab92516), anti‐CD8‐PE antibody (eBioscience, 12‐0081‐82), anti‐PD‐1‐FITC antibody (eBioscience, 11‐9981‐81) or anti‐TIM‐3 rabbit antibody (Abclonal, A2516). And the secondary antibody is Alexa Fluor 647‐conjugated antirabbit goat antibody. The slices were then visualized by CLSM. And the TUNEL staining was conducted by following the instruction of TUNEL kit.

### Safety Evaluation

For the safety evaluation, lung metastatic mice were sacrificed after being treated for five times. The blood was obtained to evaluate the level of alanine transaminase (ALT), aspartate transaminase (AST), blood urea nitrogen (BUN), and creatinine (CRE) and the hematoxylin and eosin staining was performed on the main organs sections and visualized with a light microscope.

### Statistical Analysis and Software

The data were expressed as mean ± standard deviation or mean ± standard error of the mean. Prism software package (PRISM 10.0; GraphPad Software) was used for all statistical analyses. Two‐tailed unpaired Student's *t*‐test was used for comparing two groups of data, and one‐way analysis of variance (ANOVA) with corresponding Tukey's multiple comparison was used for multiple groups of data. (*p < 0.05, **p < 0.01, and ***p < 0.001). The statistical details of the experiments are included in the figure captions.

## Conflict of Interest

The authors declare no conflict of interest.

## Supporting information



Supporting Information

Supplemental Video 1

Supplemental Video 2

## Data Availability

The data that support the findings of this study are available from the corresponding author upon reasonable request.
