# Supplementary material for: A Methionine Allocation Nanoregulator for the Suppression of Cancer Stem Cells and Support to the Immune Cells by Epigenetic Regulation
Source: Adv Sci (Weinh). 2025 Feb 22;12(15):2415207. doi: 10.1002/advs.202415207 (PMC12005795; doi:10.1002/advs.202415207)
Supplement: Supplementary file 1 — Supporting Information [file ADVS-12-2415207-s003.pdf]

ADVANCED SCIENCE
Open Access

Supporting Information

for *Adv. Sci.*, DOI 10.1002/advs.202415207

A Methionine Allocation Nanoregulator for the Suppression of Cancer Stem Cells and Support to the Immune Cells by Epigenetic Regulation

Boyu Su, Qinjun Chen, Xuwen Li, Mingzhu Fang, Yu Wang, Haolin Song, Haoyu You, Zheng Zhou, Yuxing Wu, Zhenhao Zhao, Yun Chen, Hongrui Fan, Chufeng Li, Chen Jiang and Tao Sun**

Supporting Information

A Methionine Allocation Nano-regulator for the Suppression of Cancer Stem Cells and Support to the Immune Cells by Epigenetic Regulation

Boyu Su[1], Qinjun Chen[1], Xuwen Li[1], Mingzhu Fang[1], Yu Wang[1], Haolin Song[1], Haoyu You[1], Zheng Zhou[1], Yuxing Wu[1], Zhenhao Zhao[1], Yun Chen[1], Hongrui Fan[1], Chufeng Li[1], Chen Jiang[1,2], Tao Sun[1,3]**

[1]Department of Pharmaceutics, School of Pharmacy, Fudan University, Key Laboratory of Smart Drug Delivery, Ministry of Education, State Key Laboratory of Medical Neurobiology and MOE Frontiers Center for Brain Science, Shanghai, 201203 China.

[2]Department of Digestive Diseases, National Regional Medical Center, Binhai Campus of the First Affiliated Hospital, Fujian Medical University, Fuzhou, 350212 China.

[3]Quzhou Fudan Institute, Quzhou, 324003 China.

Email: *jiangchen@shmu.edu.cn (C. Jiang) and sunt@fudan.edu.cn (T. Sun).*

Supplementary Figures

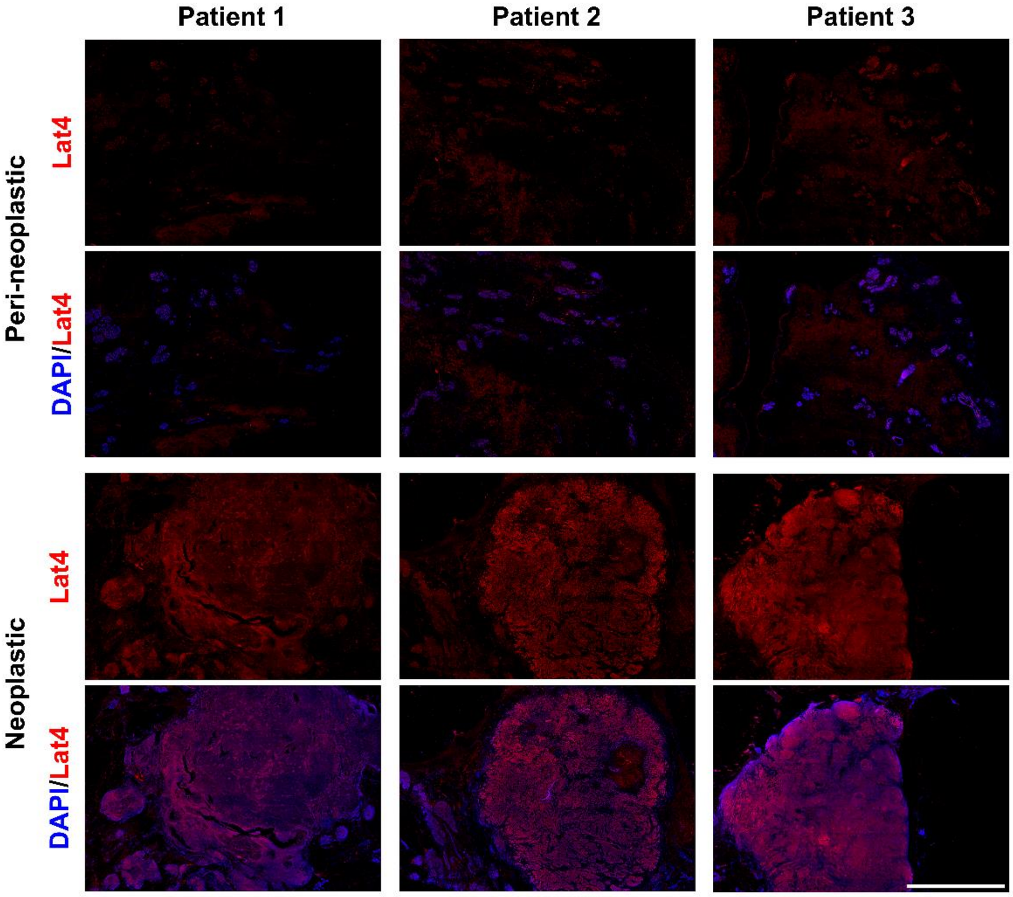

Figure S1. Lat4 expression in peri-neoplastic and neoplastic tissues from different breast cancer patients by immunofluorescence staining. Scale bar=5 mm.

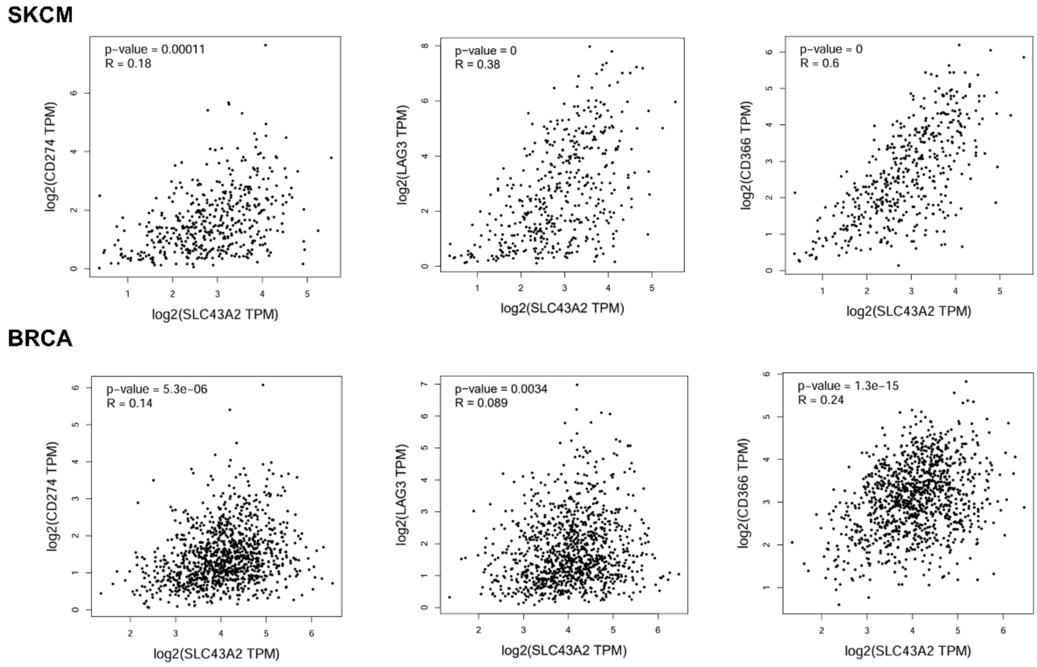

Figure S2. Correlation analysis between Lat4 (SLC43A2) and early (CD274), intermediate (LAG3) and late (CD366) markers of T cell exhaustion in SKCM patients and BRCA patients (GEPIA).

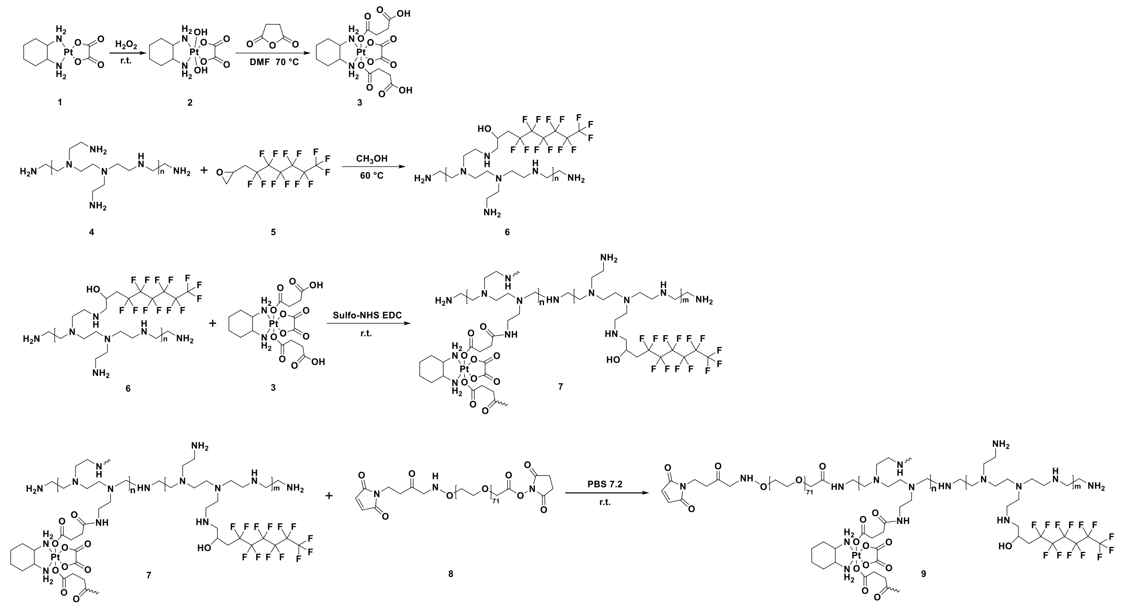

Figure S3. Synthetic route of PEG-Pt-F-PEI.

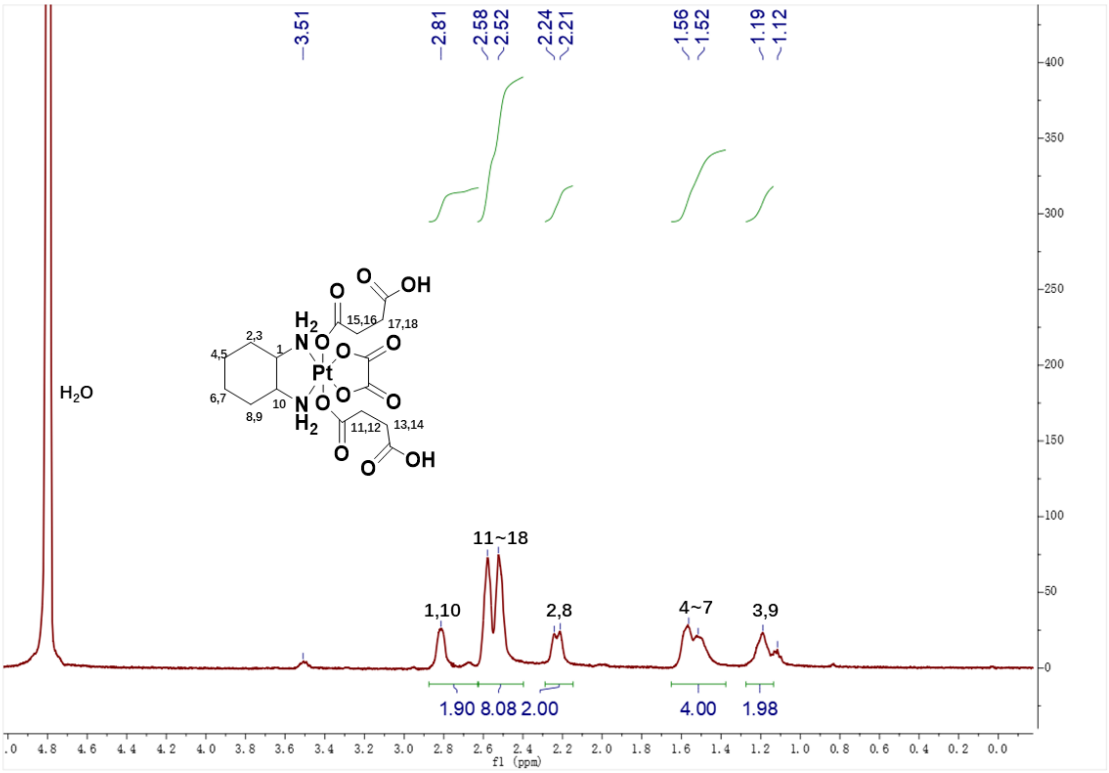

Figure S4. The ^{1}H-NMR spectrum of compound 3 in D_2O.

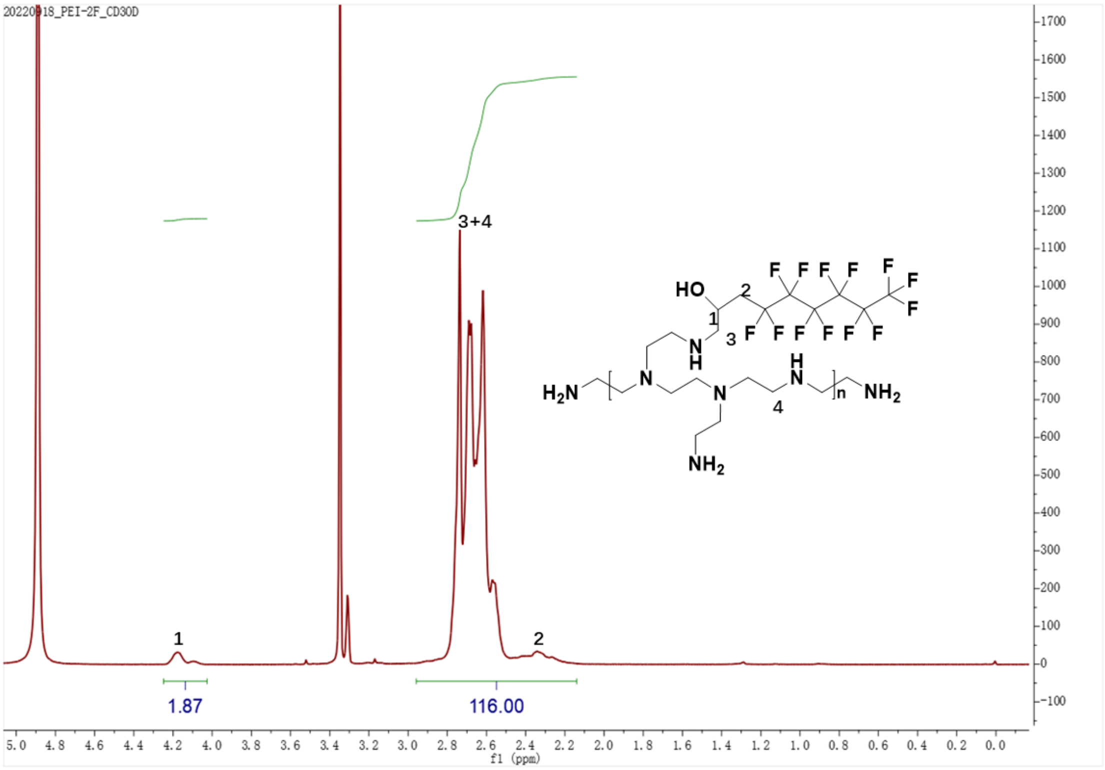

Figure S5. The ^{1}H-NMR spectrum of compound 6 in CD_3OD.

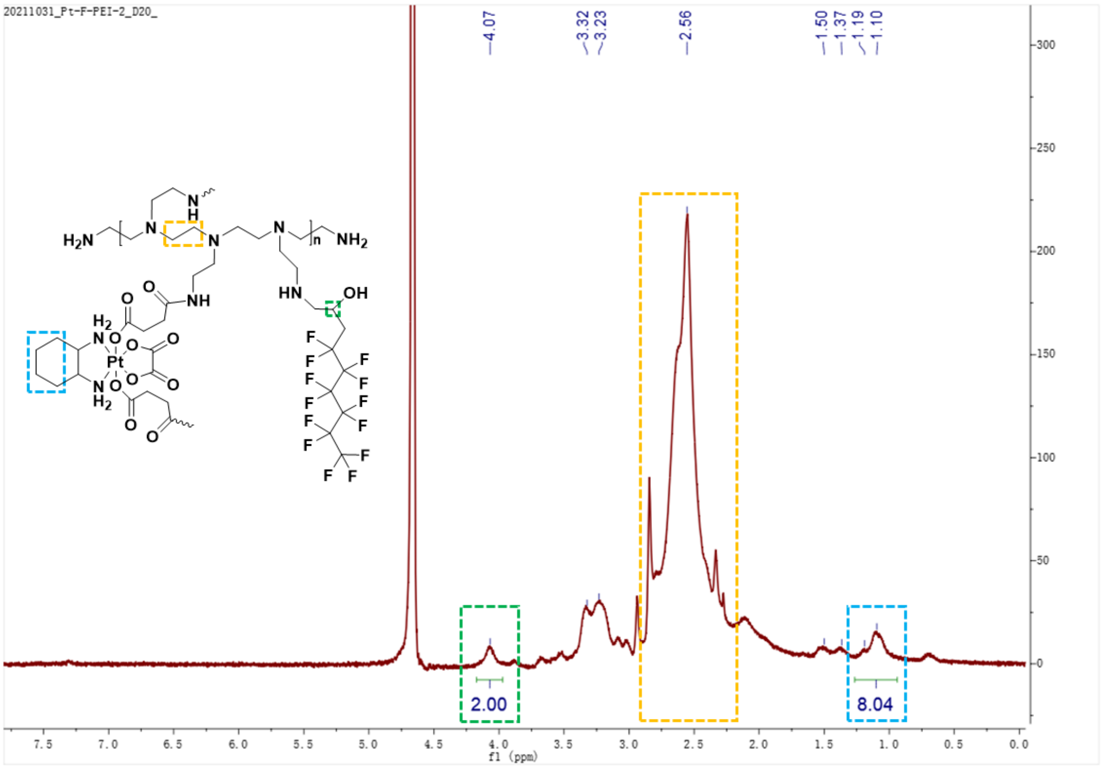

Figure S6. The ^{1}H-NMR spectrum of compound 7 in D_2O.

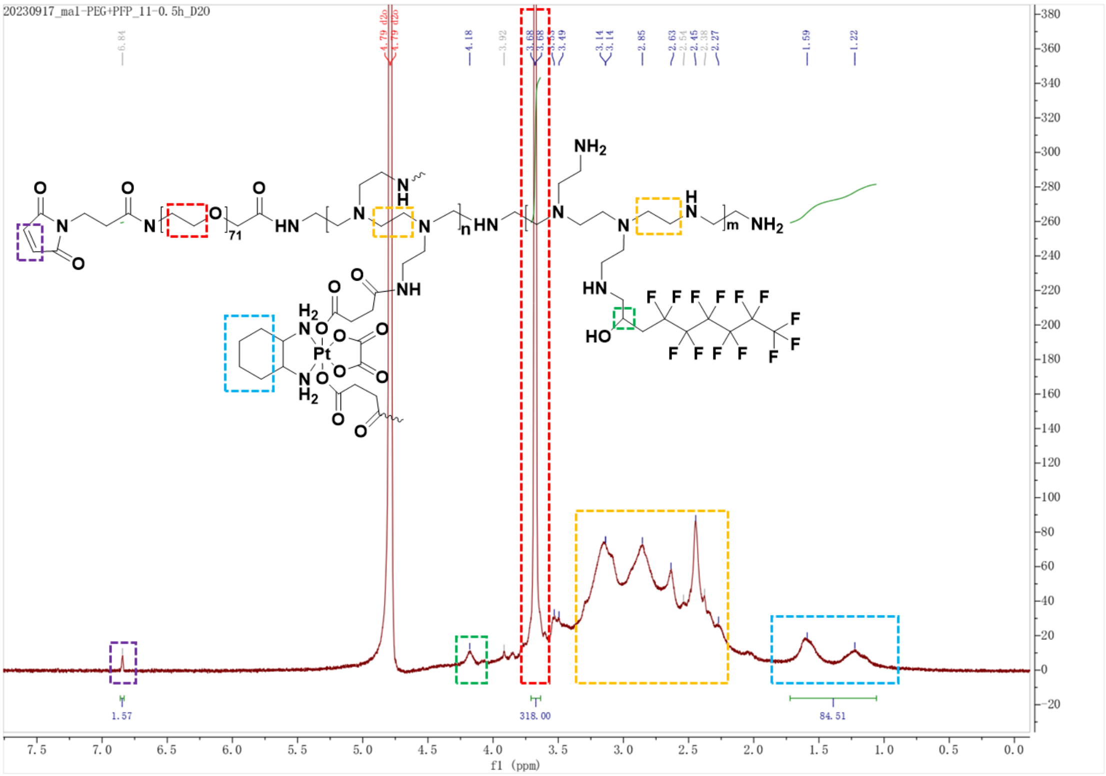

Figure S7. The 1H-NMR spectrum of compound 9 in D2O.

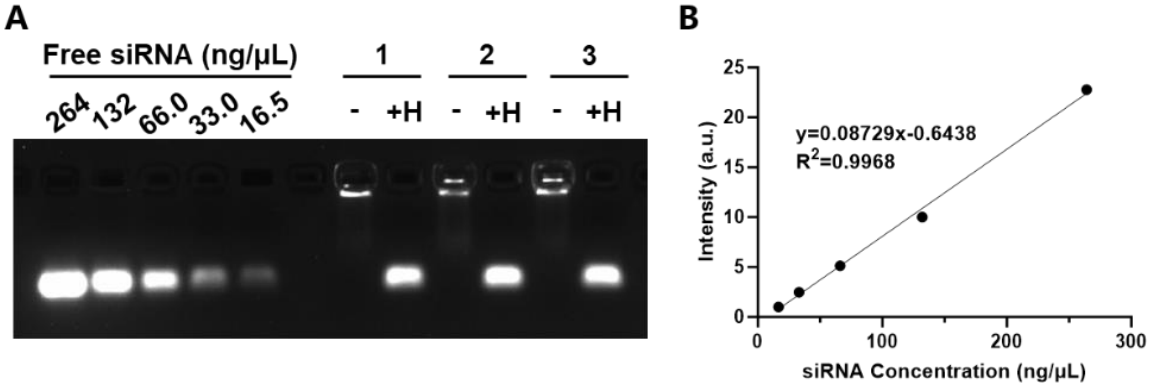

Figure S8. Determination of siRNA encapsulation efficiency in AS-F-NP. (A) Agarose gel electrophoresis images of free siRNA at gradient concentrations, along with parallel repeats of AS-F-NP without or with 2 mg/mL heparin sodium added. (B) siRNA standard curve used for quantitative calculation of encapsulation efficiency.

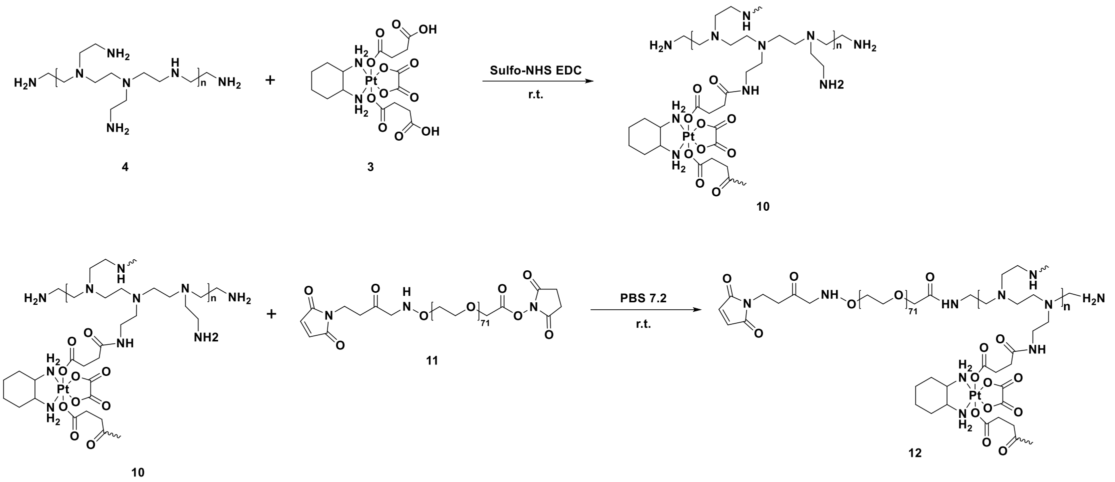

Figure S9. Synthetic route of PEG-Pt-PEI.

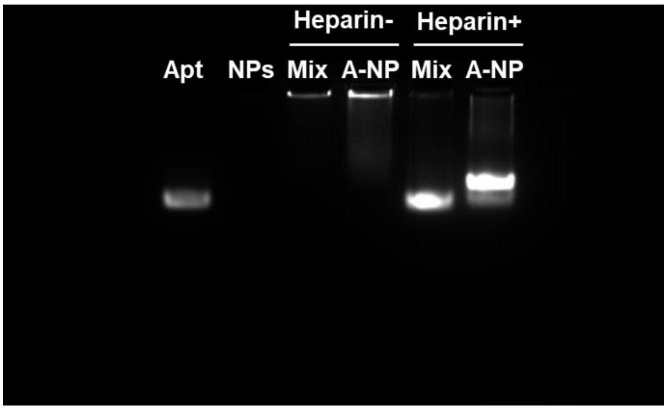

Figure S10. Agarose gel electrophoresis fluorescent image characterizing the covalent coupling of FAM-Aptamer with mal-PEG-Pt-PEI. (Channel: 488/520)

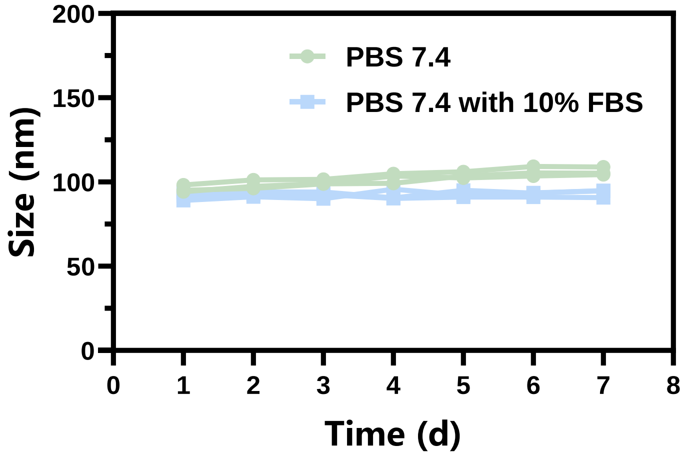

Figure S11. Stability evaluation of AS-F-NP by DLS monitoring.

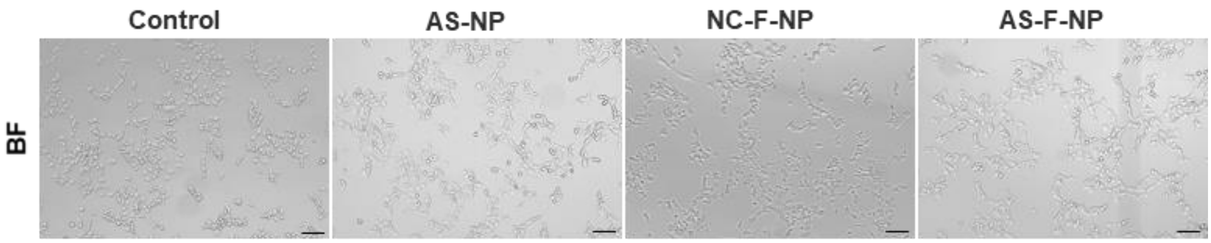

Figure S12. Bright field images of the fluorescence microscopy in Figure 2A. (AS-NP, fluorine-free nanoparticles; NC-F-NP, scramble-aptamer surface modified fluorinated nanoparticles; AS-F-NP, AS1411 surface modified fluorinated nanoparticles; scale bar = 100 μm).

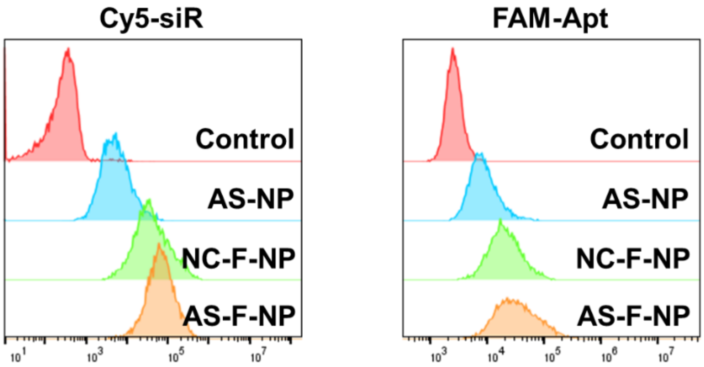

Figure S13. FACS analysis of 4T1 cell uptake of different nanoparticles (AS-NP, fluorine-free nanoparticles; NC-F-NP, control aptamer surface modified fluorinated nanoparticles; AS-F-NP, AS1411 surface modified fluorinated nanoparticles).

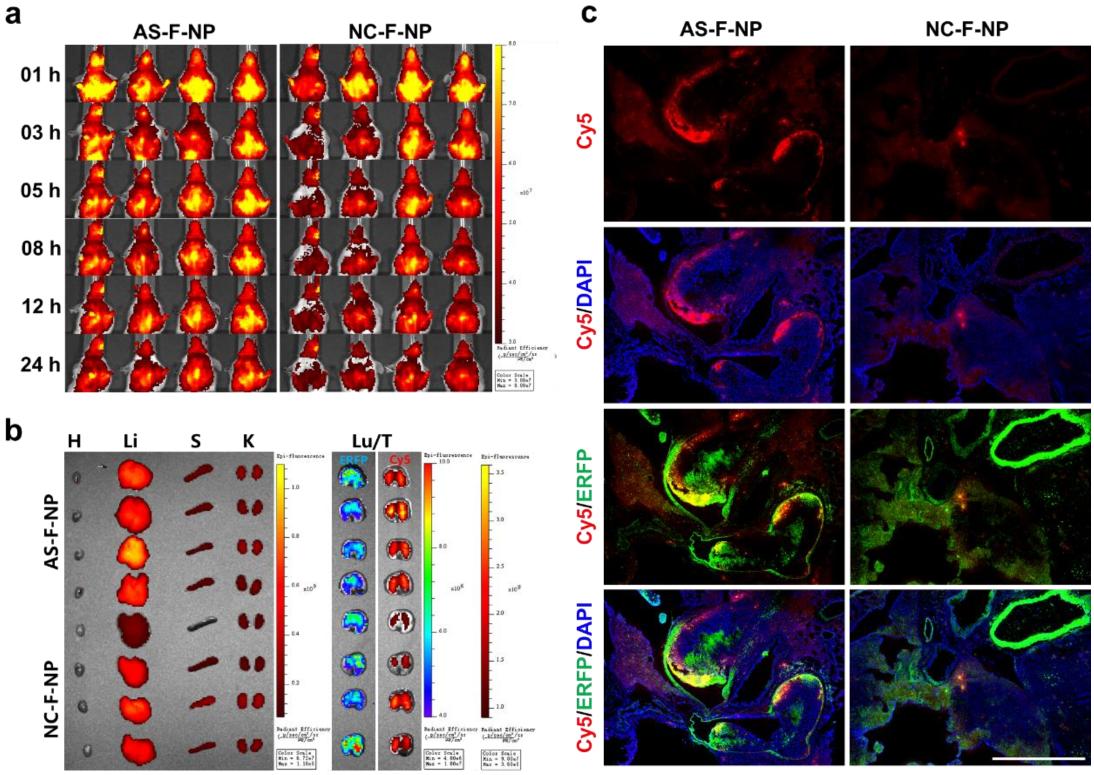

Figure S14. (a) IVIS images of 4T1 lung metastasis model mice at different time points after injected with Cy5 labeled nanoparticles. (b) *Ex vivo* IVIS images of main organs after intravenous administration of nanoparticles for 24 h. (c) Distribution of Cy5 labeled

nanoparticles in 4T1 lung metastatic nodules (scale bar= 1 mm; Blue: DAPI for nucleus; green: ERFP for 4T1 cells; red: Cy5 for nanoparticles).

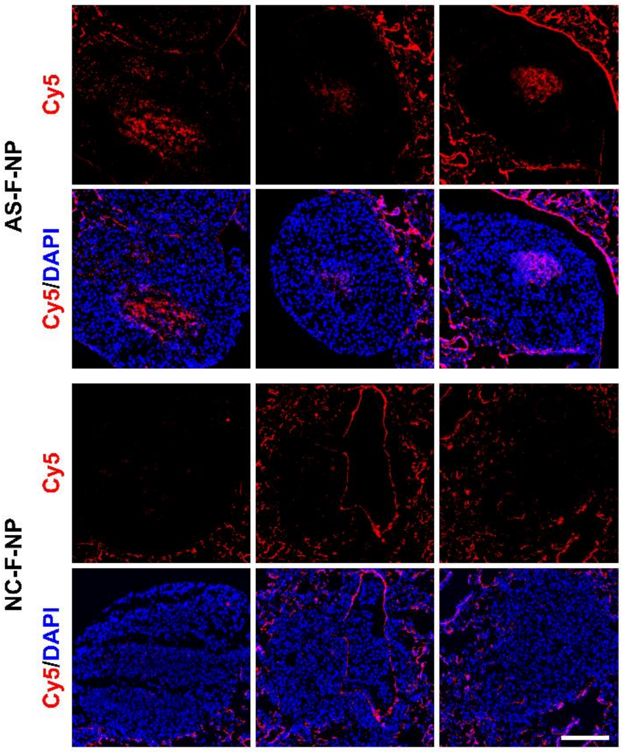

Figure S15. Distribution of Cy5 labeled nanoparticles in B16-F10 lung metastatic nodules (scale bar= 200 μm; Blue: DAPI for nucleus; red: Cy5 for nanoparticles).

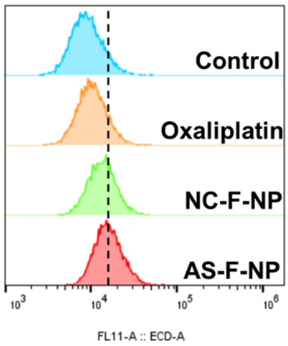

Figure S16. FACS analysis of intracellular ROS in B16-F10 cells treated with different formulations.

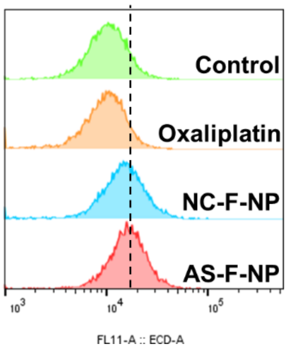

Figure S17. FACS analysis of intracellular ROS in 4T1 cells treated with different formulations.

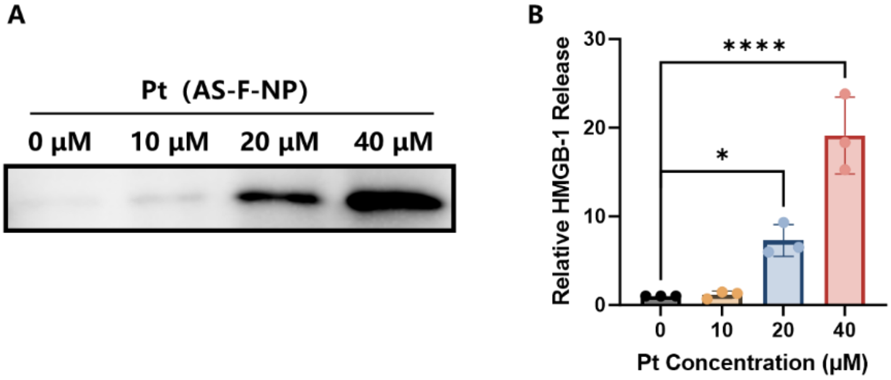

Figure S18. Representative Western Blot image (A) and quantitative results (B) of HMGB1 release in the cell supernatants of B16F10 cells treated with varying concentrations of AS-F-NP/siLat4.

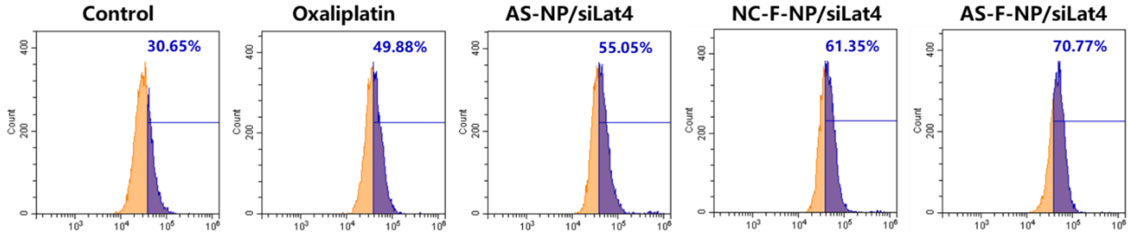

Figure S19. FACS analysis of CRT exposure on the membrane of 4T1 cells treated with different formulations.

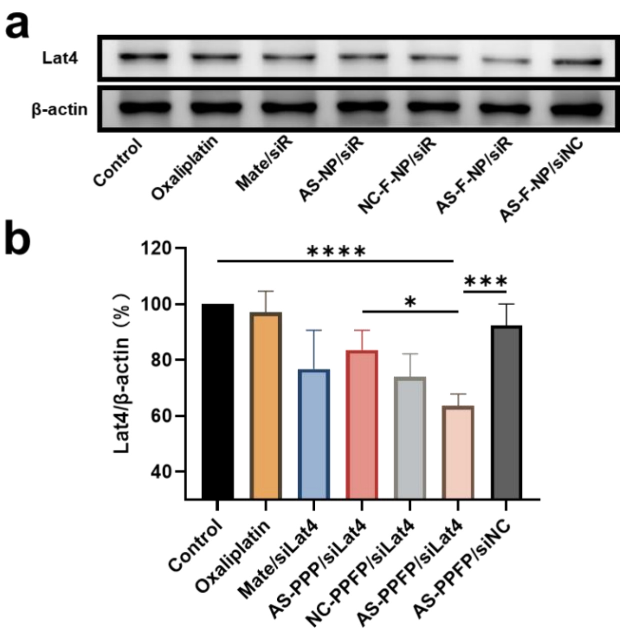

Figure S20. (a) Western blot assay and (b) semi-quantification analysis of Lat4 in 4T1 cells after treated with different formulations. The data are represented as mean ± SD (n = 3).

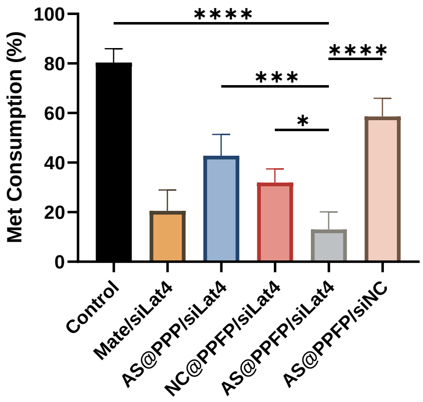

Figure S21. Methionine consumption rate of 4T1 cells after different treatments compared to fresh medium. The data are represented as mean ± SD (n = 4).

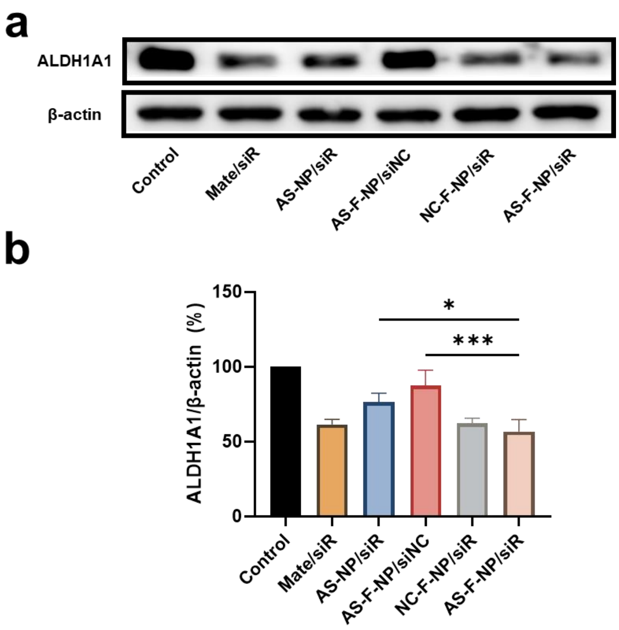

Figure S22. (a) Western blot assay and (b) semi-quantification analysis of ALDH1A1 in 4T1 cells after treated with different formulations (The same internal control image is shown in Figure 4L (main text) and Figure S22 (Supporting Information) for consistency, as the samples were run on the same gel under identical conditions). The data are represented as mean ± SD (n = 3).

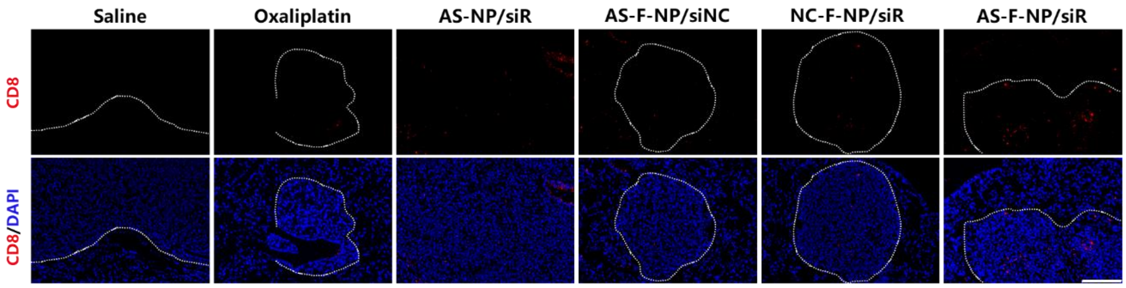

Figure S23. Immunofluorescence imaging of the infiltration of CD8+ T cells in B16-F10 lung metastatic nodules in mice with different treatment. Scale bar=200 μm.

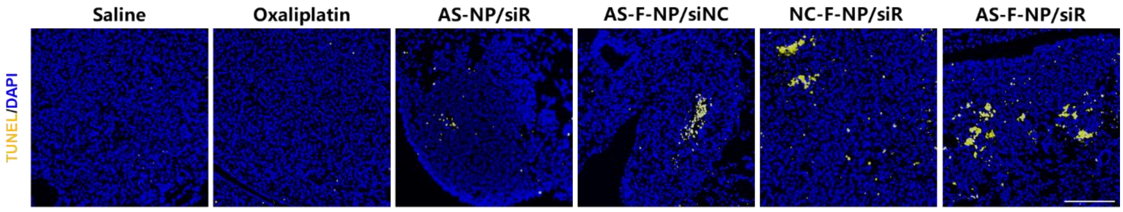

Figure S24. TUNEL staining images of B16-F10 lung metastatic nodules in mice with different treatment. Scale bar=200 μm.

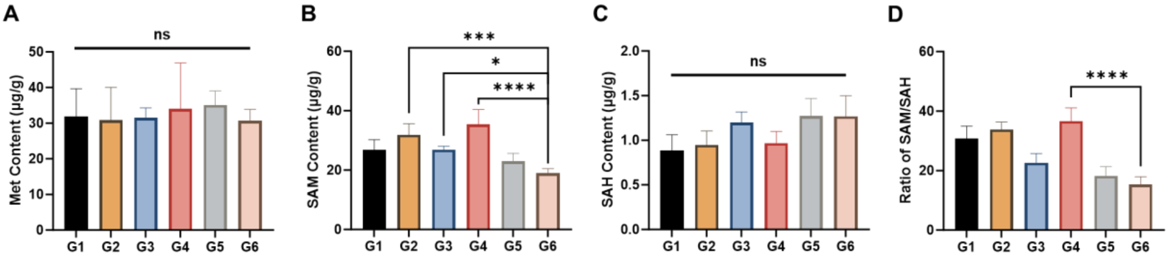

Figure S25. Evaluation of methionine consumption *in vivo*. Levels of (A) methionine, (B) S-adenosyl-methionine (SAM), and (C) S-adenosyl-homocysteine (SAH) in tumor-containing lung tissues of mice determined by LC-MS/MS and (D) the ratio of SAM to SAH used to evaluate the availability of reactive methyl groups.

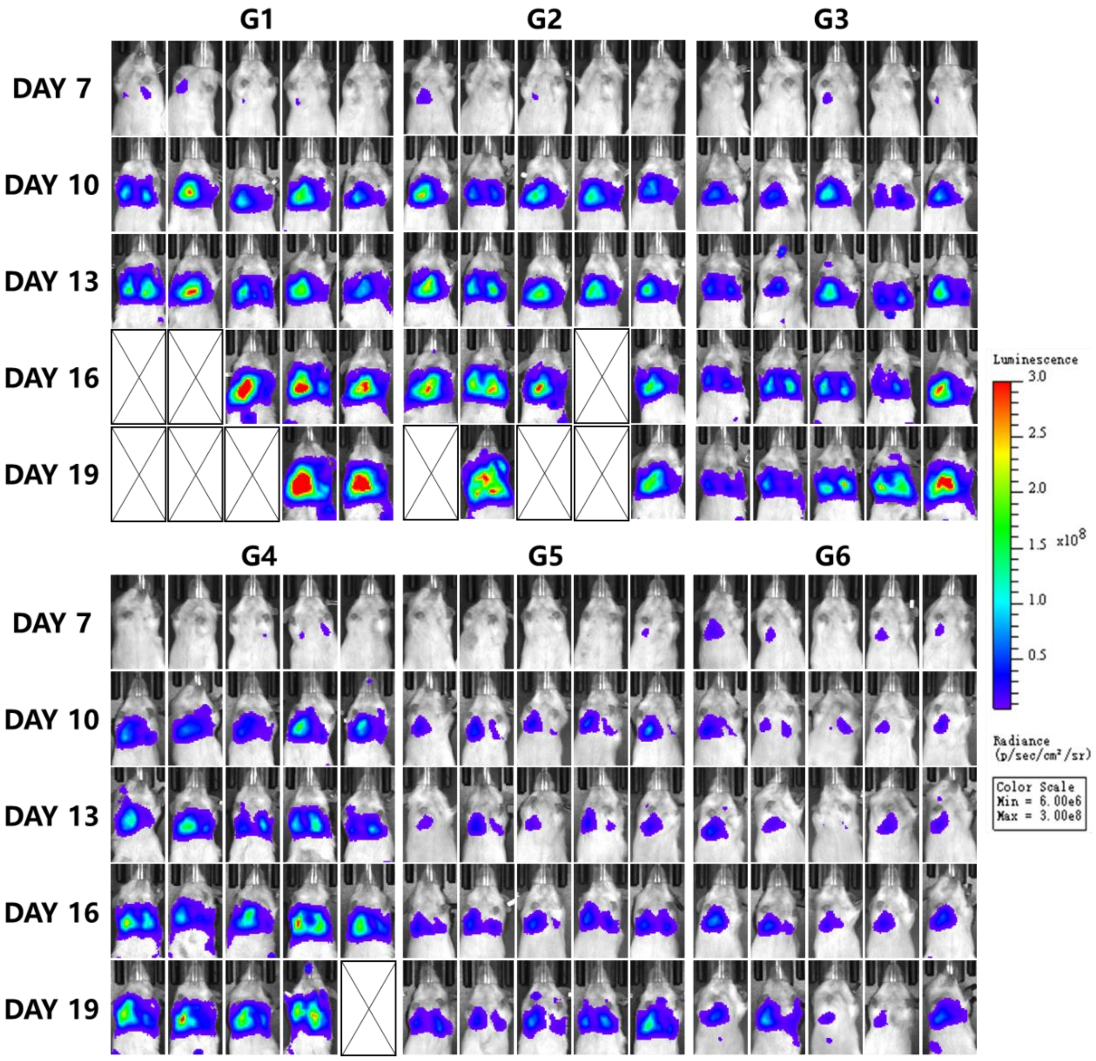

Figure S26. IVIS images of 4T1-Luc lung metastasis tumor models treated with different formulation at different time through bioluminescence detection (n=5).

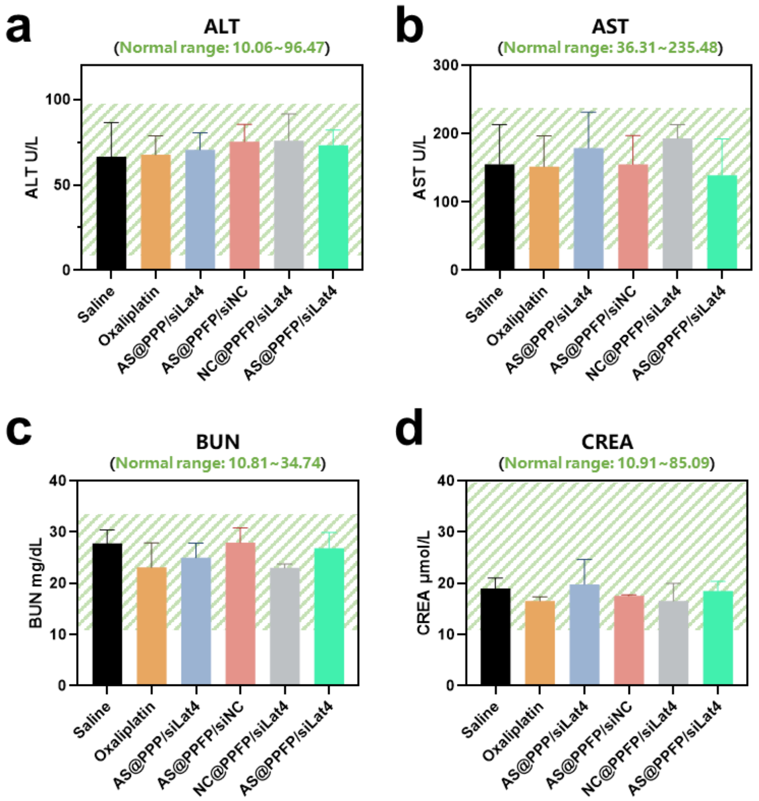

Figure S27. Liver enzyme levels (AST and ALT) and kidney function indicators (CR and BUN) in mice treated with different formulations. The data are represented as the mean ± SD (n = 3). The green shading indicates the normal ranges of each indicator. AST, aspartate aminotransferase; ALT, alanine aminotransferase; CR, serum creatinine; BUN, blood urea nitrogen.

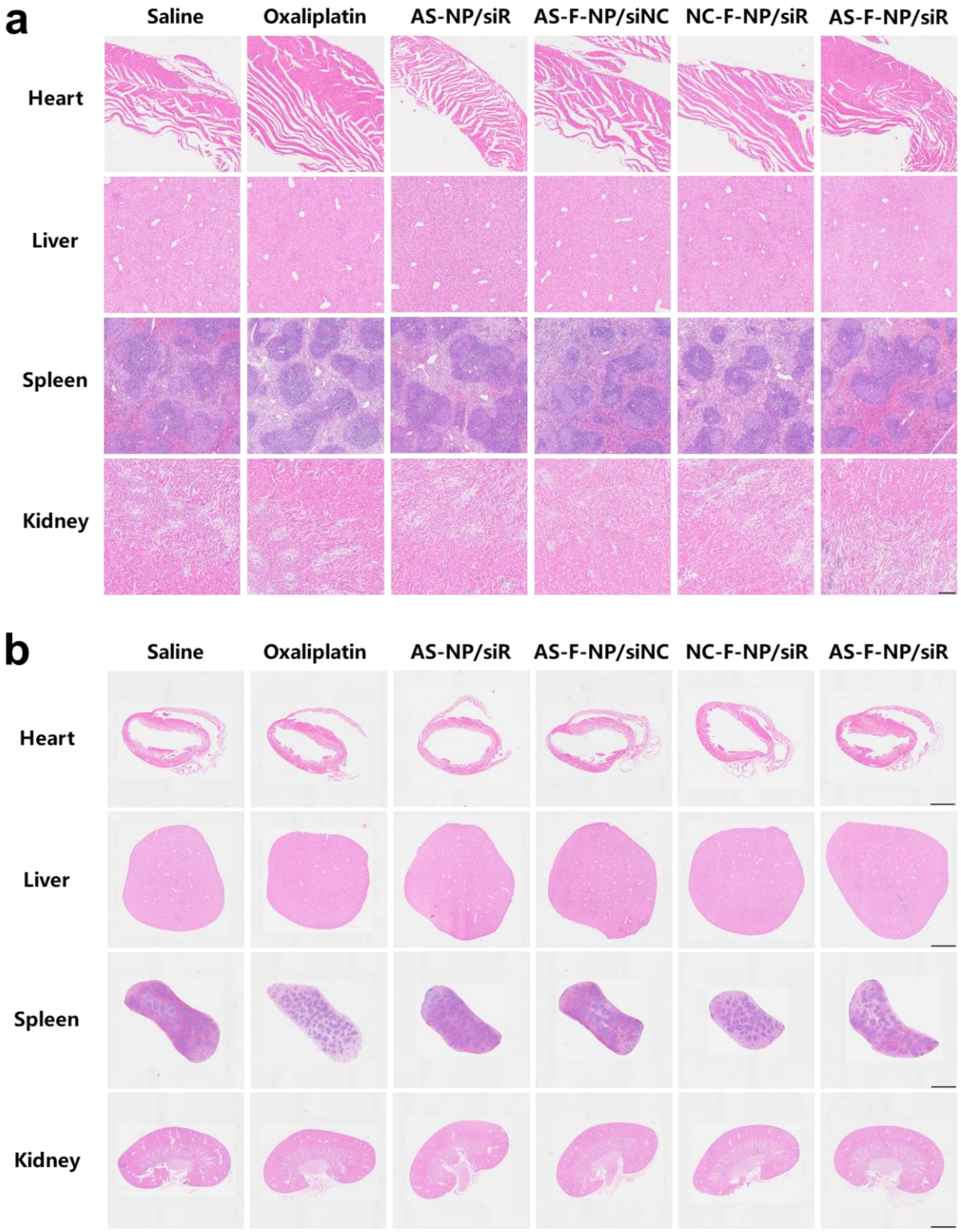

Figure S28. (a) Representative images of H&E staining of major organs (heart, liver, spleen and kidney) from B16-F10 lung metastasis model mice treated with different formulations. Scale bar = 200 μm (b) Slide scan images of H&E staining of major organs (heart, liver, spleen and kidney) from B16-F10 lung metastasis model mice treated with different formulations. Scale bar = 2 mm

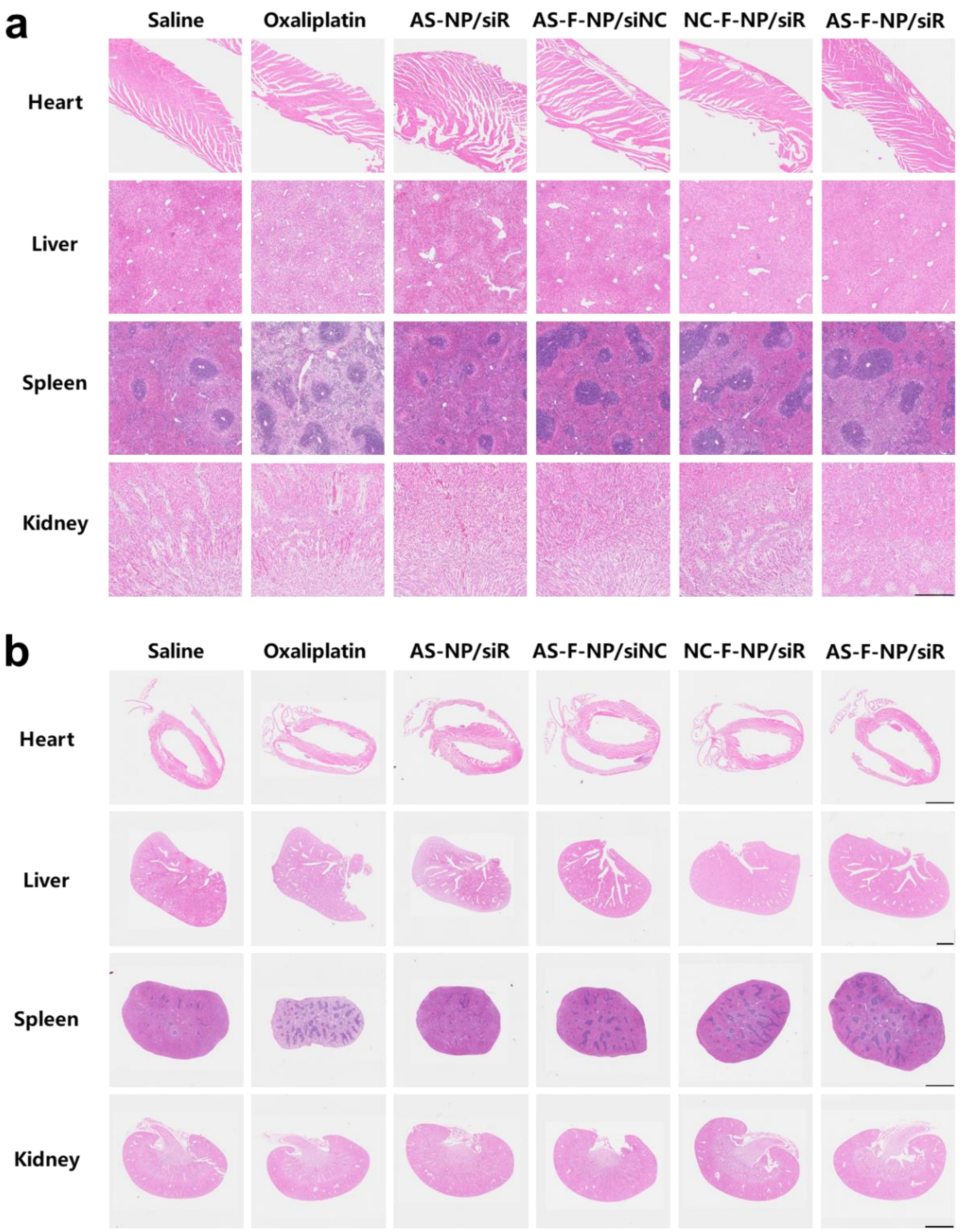

Figure S29. (a) Representative images of H&E staining of major organs (heart, liver, spleen and kidney) from 4T1 lung metastasis model mice treated with different formulations. Scale bar = 200 μm (b) Slide scan images of H&E staining of major organs (heart, liver, spleen and kidney) from 4T1 lung metastasis model mice treated with different formulations. Scale bar = 2 mm

Captions of Videos in Supporting Files:

Video S1: A series of time-lapse confocal imaging of the processes after AS-F-NP entry into the B16-F10 cells (blue: nucleus, green: lysosomes, Red: Cy5-siRNA). Images were captured per 3 min with excitation of 405/488/640 nm for a total period of 6 h.

Video S2: A series of time-lapse confocal imaging of the processes after AS-F-NP entry into the B16-F10 cells (blue: nucleus, green: lysosomes, Red: Cy5-siRNA). Images were captured at the plane of the translational Z-axis per 3 min with excitation of 405/488/640 nm for a total period of 1 h.
